# Jagged1 intracellular domain/SMAD3 complex transcriptionally regulates TWIST1 to drive glioma invasion

**DOI:** 10.1038/s41419-023-06356-0

**Published:** 2023-12-13

**Authors:** Jung Yun Kim, Nayoung Hong, Sehyeon Park, Seok Won Ham, Eun-Jung Kim, Sung-Ok Kim, Junseok Jang, Yoonji Kim, Jun-Kyum Kim, Sung-Chan Kim, Jong-Whi Park, Hyunggee Kim

**Affiliations:** 1 Department of Biotechnology, College of Life Sciences and Biotechnology, Korea University, Seoul, 02841 Republic of Korea; 2 Institute of Animal Molecular Biotechnology, Korea University, Seoul, 02841 Republic of Korea; 3 MEDIFIC Inc., Hwaseong-si, Gyeonggi-do 18469 Republic of Korea; 4 Department of Biochemistry, College of Medicine, Hallym University, Chuncheon, 24252 Republic of Korea; 5 Department of Life Sciences, Gachon University, Incheon, 21999 Republic of Korea

**Keywords:** CNS cancer, Cell invasion

## Abstract

Jagged1 (JAG1) is a Notch ligand that correlates with tumor progression. Not limited to its function as a ligand, JAG1 can be cleaved, and its intracellular domain translocates to the nucleus, where it functions as a transcriptional cofactor. Previously, we showed that JAG1 intracellular domain (JICD1) forms a protein complex with DDX17/SMAD3/TGIF2. However, the molecular mechanisms underlying JICD1-mediated tumor aggressiveness remains unclear. Here, we demonstrate that JICD1 enhances the invasive phenotypes of glioblastoma cells by transcriptionally activating epithelial-to-mesenchymal transition (EMT)-related genes, especially TWIST1. The inhibition of TWIST1 reduced JICD1-driven tumor aggressiveness. Although SMAD3 is an important component of transforming growth factor (TGF)-β signaling, the JICD1/SMAD3 transcriptional complex was shown to govern brain tumor invasion independent of TGF-β signaling. Moreover, JICD1-TWIST1-MMP2 and MMP9 axes were significantly correlated with clinical outcome of glioblastoma patients. Collectively, we identified the JICD1/SMAD3-TWIST1 axis as a novel inducer of invasive phenotypes in cancer cells.

## Introduction

Notch signaling, an evolutionarily conserved signaling pathway, plays a central role in development and tissue homeostasis [1–3]. Notch signaling is dependent on cell-cell interactions, where transmembrane receptors on one cell are activated by transmembrane ligands on juxtaposed cells [3]. Notch signaling regulates the maintenance of the stem cell pool, cell fate decisions during development, cell proliferation, and apoptosis in a cell context-dependent manner [2, 4]. Notch signaling dysregulation contributes to various diseases, including tumorigenesis and progression of various types of cancer [1, 3].

In mammalian cells, there are four different NOTCH receptors (NOTCH1-4) and five corresponding ligands (delta-like ligand 1 [DLL1], DLL3, DLL4, Jagged-1 [JAG1], and JAG2). Among the ligands, JAG1 is highly expressed in tumors and thus associated with poor prognosis in patients [5, 6]. JAG1 regulates cell proliferation, survival, and chemotherapeutic resistance in various types of cancers [7–9]. In addition, JAG1 profoundly affects cancer cell invasion and metastasis [10, 11].

Upon ligand binding, the NOTCH receptor undergoes a conformational change and is sequentially cleaved by metalloproteinase (ADAM) and γ-secretase [2, 4]. The NOTCH intracellular domain is thus translocated into the nucleus and regulates gene transcription with the centromere-binding protein 1/Suppressor of hairless/Lag1 and mastermind-like protein [2]. Several studies have revealed that the NOTCH ligand can release its intracellular domain (ICD), which is cleaved by ADAM and γ-secretase [12, 13]. In tumors, JAG1 intracellular domain (JICD1) formation is enhanced by oncogenic signaling, including the interleukin-4 (IL-4)-phosphatidylinositol 3-kinase d (PI3Kd)/protein kinase B (AKT) or Kirsten rat sarcoma virus (KRAS)/extracellular signal-regulated kinase (ERK)/ADAM17 signal [14, 15]. Furthermore, our previous study showed that JICD1 enhances tumorigenesis by forming a transcriptional complex with DEAD-box helicase 17 (DDX17)/SMAD family member 3 (SMAD3)/TGFβ-induced factor homeobox 2 (TGIF2) [16].

Glioblastoma (GBM) is a common malignant central nervous system cancer and a grade IV tumor according to the World Health Organization classification based on its histopathological characteristics [17]. Several histopathological features define GBM, including intratumoral heterogeneity, necrotic regions with pseudopalisading cells, microvascular hyperplasia, and local invasion [17].

Among these characteristics, invasion of GBM cells into the adjacent brain parenchyma interferes with complete tumor resection [18]. During invasion, cancer cells gain migratory phenotypes via epithelial-to-mesenchymal transition (EMT) [18]. EMT is a cellular mechanism by which polarized epithelial cells undergo malignant transformation and acquire mesenchymal phenotypes [19, 20]. During EMT, multiple molecular programs are altered, including transcriptional factor activation, expression of specific markers, and production of extracellular matrix-degrading proteins [19, 20]. In many types of cancer, EMT-inducing signals, such as TGF-β, regulate EMT-transcription factors (TFs) and promote the EMT process [19]. The EMT process is coordinated by EMT-TFs, such as TWIST, SNAIL, SLUG, and ZEB; these TFs alter the molecular programs related to cell migration [19, 21, 22].

In our previous study, JICD1 enhanced invasiveness in vitro and in vivo, independent of NOTCH signaling. Herein, we investigated the mechanism of the JICD1-induced invasion of glioma cells. Our data indicated that JICD1 promotes cell migration and invasion via the JICD1/SMAD3-TWIST1 axis. Moreover, transforming growth factor (TGF)-β signaling, which is a major mediator of EMT, is irrelevant to JICD1-induced invasive phenotypes.

## Results

### JAG1-derived JICD1 increases cell migration and invasion in glioblastoma

JAG1 is highly expressed in various cancer types and correlates with cancer invasiveness [5, 6, 10, 11]. We first analyzed RNA sequencing (RNA-seq) data of JAG1-overexpressing Ink4a/Arf^−/−^ mouse astrocytes (Supplementary Fig. 1A). Among the genes upregulated upon JAG1 expression, we identified 250 out of 385 genes related to cell migration.

**Fig. 1:**
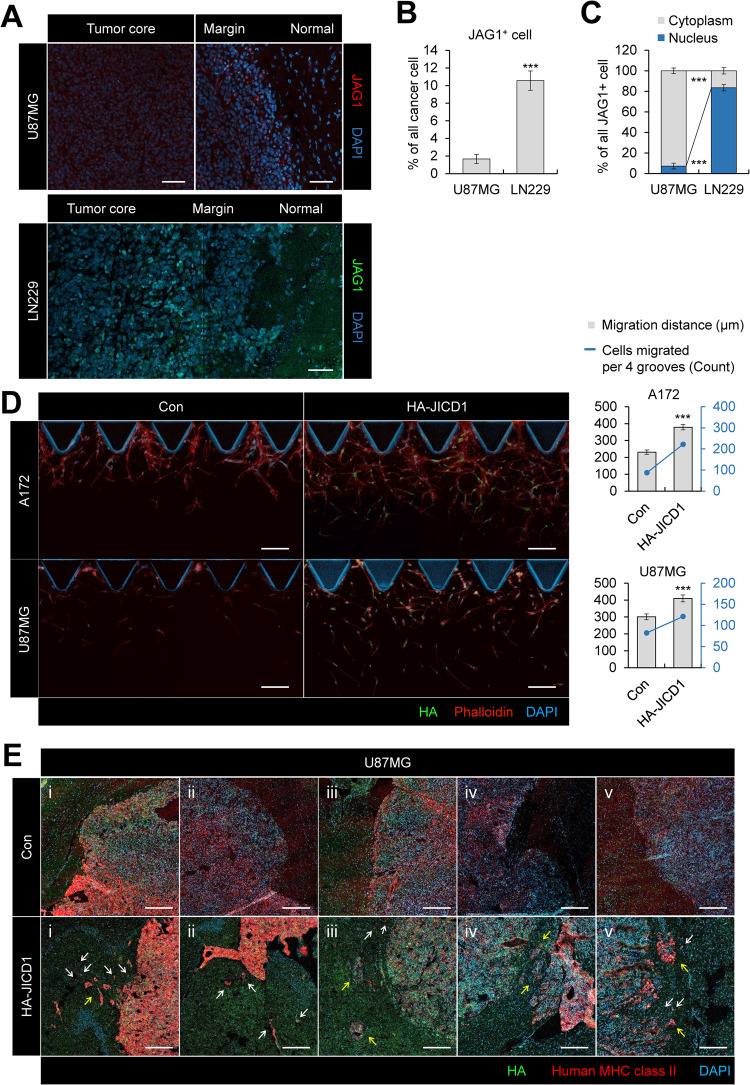
JICD1 derived from JAG1 induces tumor invasiveness. **A** Immunofluorescence showing endogenous JAG1 expression in mouse brain sections after intracranial injections of U87MG and LN229. Scale bar = 100 µm. **B** Bar graph showing the percentage of JAG1 positive cells in eight different margin regions in mouse brain sections after intracranial injections of U87MG and LN229. ***p < 0.001. **C** Bar graph showing the percentage of nuclear JAG1 in eight different margin regions in mouse brain sections after intracranial injections of U87MG and LN229. The data are presented as mean ± S.E.M. ***p < 0.001. **D** Immunofluorescence showing HA-JICD1 expression of migrated cells in A172 and U87MG control and HA-JICD1-overexpressing cells using 3D cell culture chips. A bar graph representing the average migration distance (black, left axis) and number of migrated cells per four grooves (blue, right axis) in A172 and U87MG control and HA-JICD1-overexpressing cells. Scale bar = 200 µm. ***p < 0.001. **E** Immunofluorescence showing transplanted U87MG control and HA-JICD1-overexpressing cells in tumor margin regions. White arrow indicates single cell migration and yellow arrow shows collective migration. Scale bar = 250 µm.

To investigate the correlation between JAG1 and tumor aggressiveness, we verified the expression of JAG1 in orthotopic xenografts derived from the GBM cell lines (LN229 and U87MG) (Fig. 1A). LN229 and U87MG are commonly used GBM cell lines in vitro and in vivo regarding their in vivo tumorigenicity and reflection of clinical characteristics of GBM [23]. LN229 cells form highly invasive tumors compared with U87MG cells, which generate tumors with clear margins [24]. LN229-derived tumor margins showed a higher frequency of cell population expressing JAG1 than those of U87MG-derived tumors (Fig. 1B). Among JAG1 positive cells, the population of cells with nuclear-localized JAG1 was enriched in LN229-derived tumors than in U87MG-derived tumors (Fig. 1C). In our previous study, JAG1 was highly expressed in human glioma patients, and some tumor cells exhibited nuclear JAG1 expression [16]. A part of the JAG1 protein, JICD1, is found in the nuclear fraction and its formation is repressed by the γ-secretase inhibitor, DAPT [16]. JAG1 and JICD1 were highly expressed in LN229 than in U87MG cells (Supplementary Fig. 2A). These results indicate that JICD1 derived from JAG1 is related to GBM invasiveness.

We also investigated the biological functions of JAG1 and JICD1 in Ink4a/Arf^−/−^ mouse astrocytes using RNA-seq data (Supplementary Fig. 1A, B). Distinct phenotypes related to cell signaling and migration were robustly enriched in JAG1- and JICD1-overexpressing cells. Our previous study showed that JICD1 transcriptionally regulated the stemness of glioma cells [16]. Thus, we investigated whether JICD1 promotes cell signaling and migration through transcriptional regulation.

To investigate the function of JICD1 in cancer cell migration, we generated JICD1-overexpressing cell lines (A172-HA-JICD1 and U87MG-HA-JICD1) (Supplementary Fig. 2B). In our previous study, glioma stem cell GSC20 highly expressed JICD1 among various GBM cell lines [16]. The expression of exogenously expressed JICD1 in A172 and U87MG was comparable to the expression level of JICD1 in GSC20, whereas higher than that of LN229. JICD1 overexpression did not increase cell proliferation (Supplementary Fig. 2C). We performed a wound closure assays using scratches generated on cell monolayers of control (A172-Con and U87MG-Con) and JICD1-overexpressing cells. Wound closure rates were found to be higher in JICD1-overexpressing cells than in the controls (Supplementary Fig. 2D). Additionally, we performed a transwell migration assay with JICD1-overexpressing cells (Supplementary Fig. 2E, top panels). The intensity of cell migrated through the porous membrane increased following JICD1 overexpression. Cell migration and invasion are distinct aspects of cell biology. Migration is the directed movement of cells on a substrate, whereas invasion entails the reorganization of the three-dimensional (3D)-matrix. To verify JICD1-induced cell migration in 3D-matrix settings mimicking invasion, we performed Matrigel invasion assay (Supplementary Fig. 2E, bottom panels). Cell migration intensity was significantly increased in JICD1-overexpressing cells. The results suggested that JICD1 induces cell migration and invasion. We also performed a 3D cell invasion assay using the 3D Cell Culture Chips (Fig. 1D). The number and migration distance of migrating cells increased in JICD1-overexpressing cells. These results suggested that JICD1 contributes to the migratory and invasive properties of glioma cells. Furthermore, intracranial injection of U87MG-Con or U87MG-HA-JICD1 cells revealed that JICD1-overexpressing tumors had more invasive margins than those of control tumors (Fig. 1E and Supplementary Fig. 3). Interestingly, both single-cell migration and collective motion of small cell cohorts were observed in the U87MG-HA-JICD1-derived tumor margins (Fig. 1E). Collectively, JICD1 enhanced the invasiveness and aggressiveness of glioma cells.

### JICD1 increases migration and invasion of glioma cells through transcriptional regulation of TWIST1-related EMT genes

**Fig. 2:**
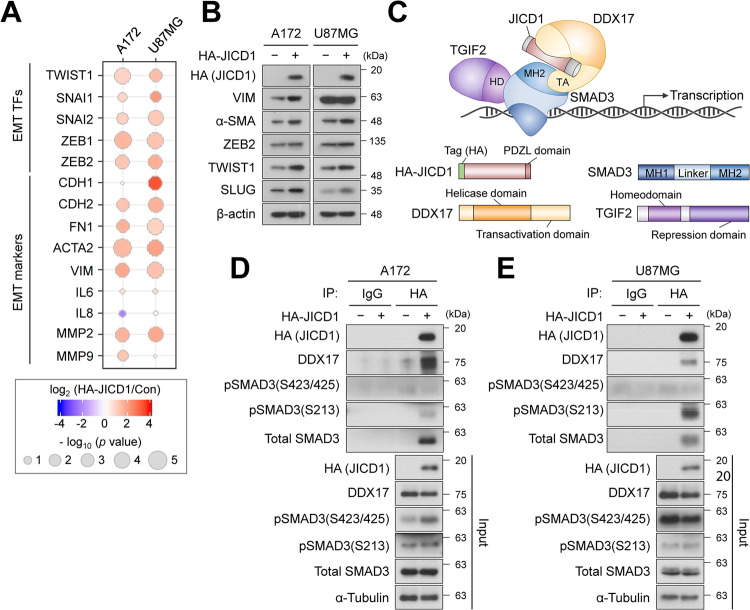
JICD1 transcriptional complex promotes gene expression related to EMT. **A** mRNA expression of EMT-TFs and markers in A172 and U87MG control and HA-JICD1-overexpressing cells. **B** Protein expression of canonical EMT-TFs and markers in A172 and U87MG control and HA-JICD1-overexpressing cells. **C** Schematic diagram that represents JICD1 transcriptional complex with DDX17, SMAD3, and TGIF2. **D**, **E** The association between ectopic HA-JICD1 and endogenous DDX17 and SMAD3 in A172 (**D**) and U87MG (**E**) control and HA-JICD1 overexpressing cells.

Our previous study clarified the function of JICD1 as a cofactor of the transcriptional complex [16]. Additionally, JAG1 regulates the expression of EMT-TFs, EMT markers, and matrix metalloproteinases (MMP2 and MMP9) [25–27]. To verify the molecular mechanism underlying JICD1-regulated cell migration and invasion, we measured the expression of canonical EMT-TFs in control and JICD1-overexpressing Ink4a/Arf^−/−^ astrocytes using RNA-seq results (Supplementary Fig. 4A). Among canonical EMT-TFs, *Twist1*, *Snai2*, and *Zeb2* were upregulated in JICD1-overexpressing Ink4a/Arf^−/−^ astrocytes. Furthermore, the mRNA expression of most canonical EMT-TFs and well-known EMT markers were increased in JICD1-overexpressing cells (Fig. 2A). Consistent with this, we observed increased protein expression of alpha-smooth muscle actin (α-SMA), vimentin (VIM), and EMT-TFs (TWIST1, SLUG, and ZEB2) in JICD1-overexpressing cells, especially that of TWIST1 (Fig. 2B). Moreover, α-SMA protein expression increased in JICD1-overexpressing cells in the 3D environment (Supplementary Fig. 4B, C). Consistent with a previous study [28], switching from E-cadherin to N-cadherin, which has been reported in classical epithelial cells during EMT, was not observed (Fig. 2A).

TWIST1, a basic helix-loop-helix TF, is a master regulator of mesodermal specification and differentiation during embryonic development [29, 30]. TWIST1 is highly expressed in various cancer cells and its expression is associated with cancer cell invasion and metastasis [31, 32]. In GBM, TWIST1 regulates the transcription of various EMT markers, such as *FN1*, *IL6*, *IL8*, *MMP2*, and *MMP9* [33–35]. Downstream target genes transcriptionally regulated by TWIST1 were upregulated in JICD1-overexpressing cells (Fig. 2A). These results suggest that JICD1 may alter the molecular repertoire related to cell motility and ECM remodeling by modulating TWIST1.

JAG1 regulates cell signaling and induces oncogenic phenotypes, such as stemness in glioma, via JICD1 formation [16]. Gene ontology analysis also showed that cell signaling regulation enriched in JAG1- and JICD1-overexpressing Ink4a/Arf^−/−^ astrocytes (Supplementary Fig. 1). As previously reported, JICD1 forms a transcriptional complex with DDX17, TGIF2, and SMAD3 (Fig. 2C). JICD1 directly binds to DDX17, and the N-terminal domain of DDX17 binds to the MAD homology 2 (MH2) domain of SMAD3. Additionally, the SMAD3 linker domain interacts with the homeodomain (HD) of TGIF2 [16]. Collectively, the JICD1 transcriptional complex binds to the promoters of target genes via SMAD3 [16]. To confirm the formation of the JICD1 transcriptional complex in JICD1-overexpressing cells, we performed immunoprecipitation (IP) with anti-HA antibody in JICD1-overexpressing cells. JICD1 was bound to DDX17 and SMAD3 in JICD1-overexpressing cells (Fig. 2D, E). Given that the SMAD3 protein has phosphorylatable sites in the C-tail of the MH2 and linker domains [36–38], we investigated the phosphorylation status of JICD1-bound SMAD3. JICD1 binds to the linker domain (Ser 213) phosphorylated SMAD3 but not to the C-tail (Ser 423/425) phosphorylated-one (Fig. 2D, E).

### JICD1-induced cell migration and invasion are distinct from TGF-β signaling

TGF-β signaling is a key effector of EMT in cancer metastasis by increasing EMT-TF expression [39, 40]. TGF-β inhibits epithelial markers and induces mesenchymal markers in various epithelial and cancer cells. SMAD3 is one of the main signal transducers in the TGF-β signaling pathway [38, 41, 42]. To confirm whether the JICD1-induced invasive phenotypes depend on TGF-β signaling, we analyzed the enrichment of TGF-β signaling gene signatures using RNA-seq data from JICD1-overexpressing Ink4a/Arf^-/-^ astrocytes (Supplementary Fig. 5A); interestingly, two TGF-β signaling gene signatures (oncogenic signature and Broad MsigDB Hallmark) were downregulated. These results indicated that genes associated with TGF-β signaling were downregulated in JICD1-overexpressing Ink4a/Arf^-/-^ astrocytes. Furthermore, TGF-β signaling-induced EMT genes were also downregulated (Supplementary Fig. 5B).

**Fig. 3:**
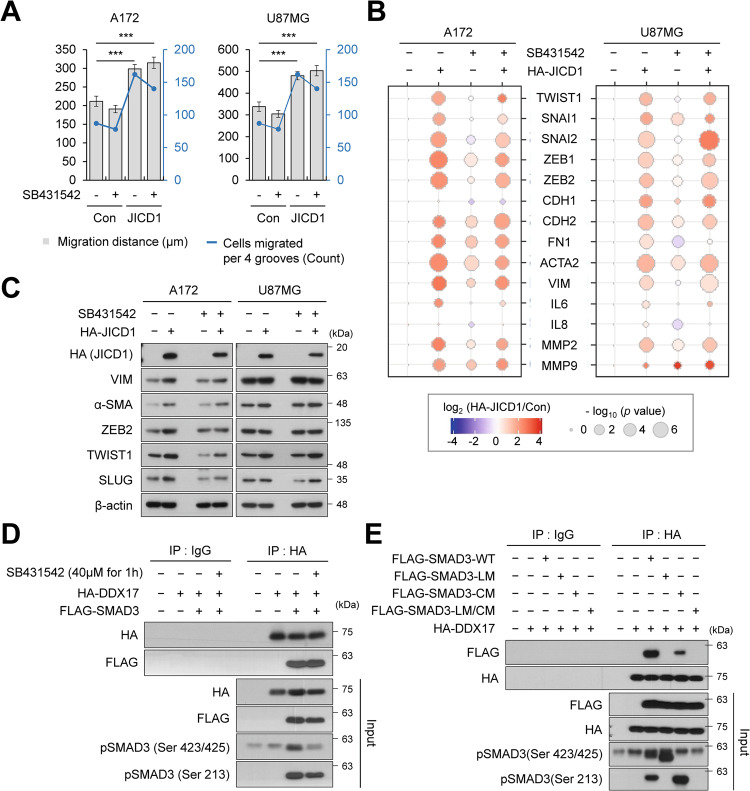
EMT induced by JICD1 is independent of TGF-β signaling. **A** Bar graph representing the average migration distance (black, left axis) and number of migrated cells per four grooves (blue, right axis) in A172 and U87MG control and HA-JICD1-overexpressing cells with SB431542 treatment (A172: 10 μM, U87MG: 20 μM). ***p < 0.001. **B** mRNA expression of EMT-related genes in A172 and U87MG control and HA-JICD1-overexpressing cells with the SB431542 treatment. **C** Protein expression of canonical EMT-TFs and markers in A172 and U87MG control and HA-JICD1-overexpressing cells with SB431542 treatment. **D** Association between HA-DDX17 and FLAG-SMAD3 in HEK293T cells with SB431542 treatment. **E** Association between HA-DDX17 and FLAG-SMAD3 mutants in HEK293T cells.

JICD1 induced cell migration and invasion by increasing TWIST1 expression (Figs 1, 2). To verify whether JICD1-mediated invasive phenotypes and expression of EMT-related genes were distinct from TGF-β signaling, we performed a 3D cell-migration assay following treatment with SB431542 (an ALK5 inhibitor) and LY2109761 (an ALK5 and TGF-β receptor II inhibitor) (Fig. 3A and Supplementary Figs 5C, 6). Although the inhibitors showed slightly different trends, the inhibition of TGF-β signaling had no effect on the migration distance or number of migrating cells. Moreover, the expression of EMT-TFs and markers was increased in JICD1-overexpressing cells compared to that in control cells, even after the inhibition of TGF-β signaling (Fig. 3B, C, Supplementary Figs. 5D, 7, 8). SB431542 and LY2109761 significantly down-regulated C-tail phosphorylation of SMAD3 and did not affect SMAD3 linker phosphorylation. LY2109761 targets TGF-β receptor II in addition to ALK5. Although both inhibitors showed similar effect on canonical TGF-β signaling, LY2109761 down-regulated the expression of EMT-TFs and markers. Nevertheless, the expression of several EMT-TFs and markers, such as TWIST1, MMP2, and MMP9, were elevated after LY2109761 treatment. These results suggest that JICD1 induces invasive phenotypes and EMT-related gene expression independent to TGF-β signaling.

Additionally, we validated the mRNA expression of key factors involved in TGF-β signaling in JICD1-overexpressing cells (Supplementary Fig. 5E). Although each cell line showed slightly different trends, most ligands and signal transducers (SMAD2, SMAD3, and SMAD4) were downregulated. There was no consistent pattern for I-SMADs (SMAD6 and SMAD7) in JICD1-overexpressing A172 and U87MG cells. Furthermore, we verified the expression and phosphorylation status of R-SMADs (SMAD2 and SMAD3) in JICD1-overexpressing cells compared with control cells (Supplementary Fig. 5F). SMAD2 phosphorylation decreased in both cell lines. C-tail phosphorylation of SMAD3 significantly increased, particularly in A172-HA-JICD1 cells. There was no difference in SMAD3 linker phosphorylation in both A172-HA-JICD1 and U87MG-HA-JICD1 cells. Total protein expression of SMAD2 did not change in either JICD1-overexpressing cell line, while the expression of SMAD3 was slightly decreased in A172-HA-JICD1 cells and increased in U87MG-HA-JCID1 cells.

To verify whether JICD1 transcriptional complex formation depends on TGF-β signaling and SMAD3 phosphorylation status, we performed IP against HA-DDX17 after treatment with SB431542 using HA-DDX17 and FLAG-SMAD3 expressing transformed human embryonic kidney 293 (HEK293T) cells (Fig. 3D). Treatment with SB431542 reduced the C-tail phosphorylation of SMAD3 without affecting the interaction between DDX17 and SMAD3. These results suggest that JICD1 indirectly regulates SMAD protein phosphorylation and that JICD1 transcriptional complex formation is distinct from TGF-β signaling.

To further understand the dependency of JICD1 transcriptional complex formation on SMAD3 phosphorylation status, we constructed point mutations of the phosphorylation site on the SMAD3 linker and C-tail (Supplementary Fig. 5G). We confirmed that the mutants in these domains failed to phosphorylate (Fig. 3E). Using these mutant proteins, we performed IP against HA-DDX17 in HEK293T cells (Fig. 3E). SMAD3 mutants with linker mutations (SMAD3-LM) or mutations in the linker and C-tail (SMAD3-LM/CM) fail to interact with DDX17. However, mutants with C-tail mutations (SMAD3-CM) showed slightly attenuated DDX17 interactions. These results demonstrate that phosphorylation of the linker domain of SMAD3 regulates interaction with DDX17. In addition, C-tail phosphorylation may regulate SMAD3 and JICD1 transcriptional complex stability [43, 44]. These findings correspond to the results that JICD1 binds to DDX17 and linker phosphorylated SMAD3 in JICD1-overexpressing cells (Fig. 2D, E).

### JICD1 transcriptional complex binds to the promoter and activates transcription of TWIST1

**Fig. 4:**
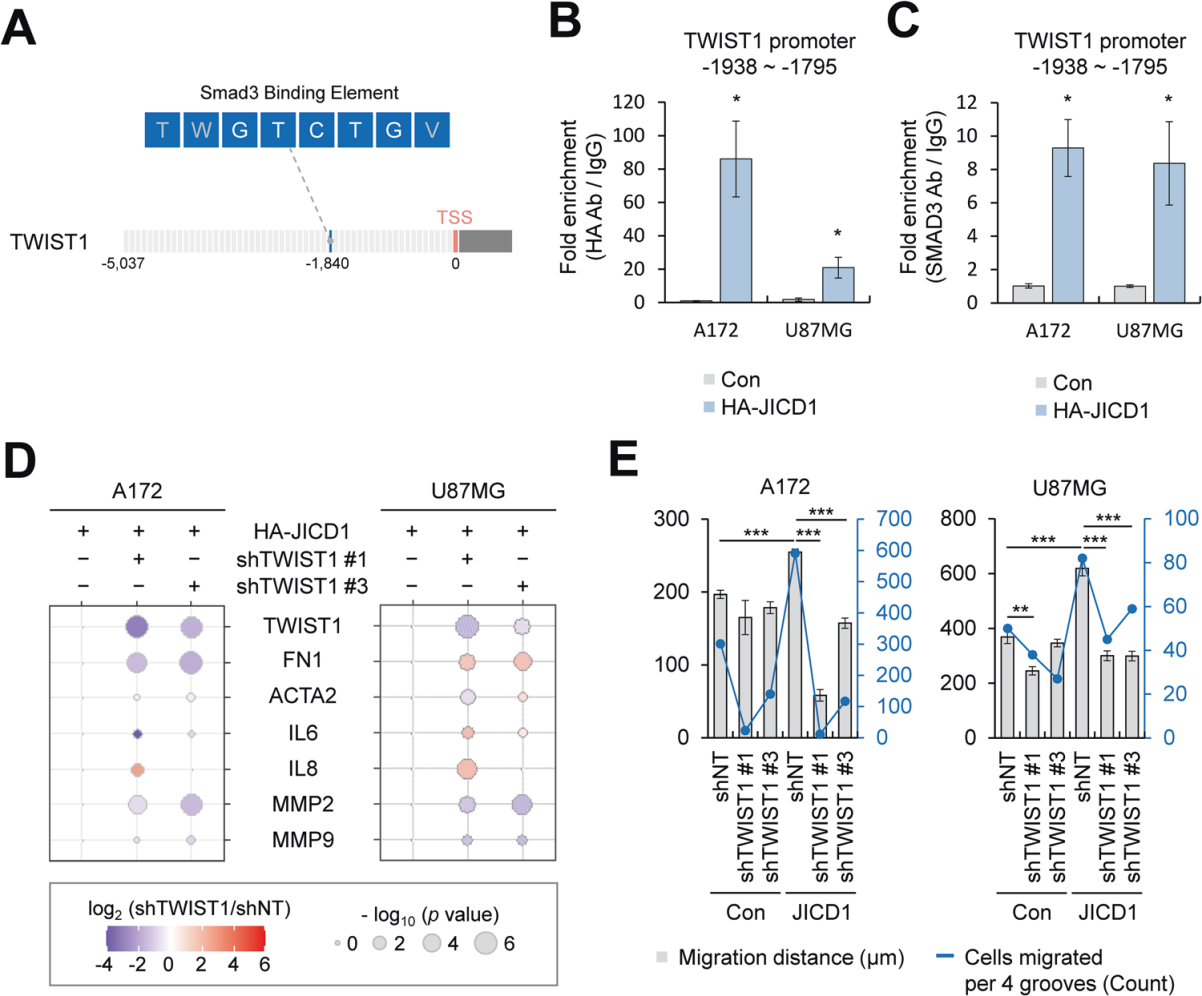
TWIST1, transcriptionally activated by JICD1 transcriptional complex, induces GBM invasion. **A** A schematic diagram of a SMAD3 binding element (TWGTCTGV) in the *TWIST1* promoter. **B**, **C** ChIP-PCR analysis of (**B**) HA-JICD1 and (**C**) SMAD3 engagement with the SMAD3 binding motif at the *TWIST1* promoter in A172 and U87MG control and HA-JICD1-overexpressing cells. *p < 0.05. **D** mRNA expression of EMT-related genes in A172 and U87MG control and HA-JICD1-overexpressing cells with TWIST1 knockdown. **E** Bar graph representing the average migration distance (black, left axis) and number of migrated cells per four grooves (blue, right axis) in A172 and U87MG control and HA-JICD1-overexpressing cells with TWIST1 knockdown. *p < 0.05, ***p < 0.001.

SMAD3 is a TGF-β signaling-related TF that binds to a DNA motif (GTCTG), called the SMAD3 binding element (SBE) [45]. Additionally, it mediates DNA binding to the JICD1 transcriptional complex [16]. Among EMT-TFs, TWIST1 and SLUG were upregulated in JICD1-overexpressing cells even after inhibiting TGF-β signaling (Fig. 3C). To investigate how the JICD1 transcriptional complex is associated with the transcriptional activation of EMT-TFs and markers, we first screened the SBE in the TWIST1 and SNAI2 promoters. There was one SBE in the TWIST1 promoter (transcription start site [TSS] −1840) and SNAI2 promoter (TSS −133) (Fig. 4A and Supplementary Fig. 9A). We confirmed that JICD1 significantly binds to TSS −1937 to −1795 on the TWIST1 promoter in JICD1-overexpressing cells using a chromatin immunoprecipitation (ChIP) assay with a hemagglutinin (HA) antibody (Fig. 4B). As JICD1 indirectly binds to DNA, we performed a ChIP assay for SMAD3, which is a DNA-binding partner in the JICD1 transcriptional complex. We found that SMAD3 was also bound to the same region in the TWIST1 promoter in both A172 and U87MG-HA-JICD1 (Fig. 4C). These results suggest that the JICD1 transcriptional complex binds to the TWIST1 promoter via SMAD3. Similarly, the JICD1 transcriptional complex was bound to TSS −162 to +13 on the SNAI2 promoter via SMAD3 in A172-HA-JICD1 but not in U87MG-HA-JICD1 (Supplementary Fig. 9B, C). In conclusion, TWIST1 is a conserved EMT-TF transcriptionally regulated by JICD1 in gliomas.

### TWIST1 silencing inhibits cell migration and invasion induced by JICD1 transcriptional complex

The JICD1 transcriptional complex transcriptionally regulated the expression of TWIST1 (Figs 2A, B, 4B, C). To confirm that TWIST1 is a key factor in JICD1-induced cell migration and invasion, we silenced TWIST1 in JICD1-overexpressing cells (Fig. 4D, Supplementary Figs 10, 11). Among the EMT markers and TWIST1 target genes, the mRNA expression of MMP2 and MMP9 was commonly downregulated in TWIST1 depleted JICD1-overexpressing cells. Thus, MMP2 and MMP9 are conserved targets of the JICD1/SMAD3-TWIST1 axis. To investigate the effect of TWIST1 silencing on the invasive phenotype of JICD1-overexpressing cells, 3D cell-invasion assay was performed. The distance and number of migrating cells decreased after TWIST1 knockdown in JICD1-overexpressing cells (Fig. 4E and Supplementary Fig. 12).

**Fig. 5:**
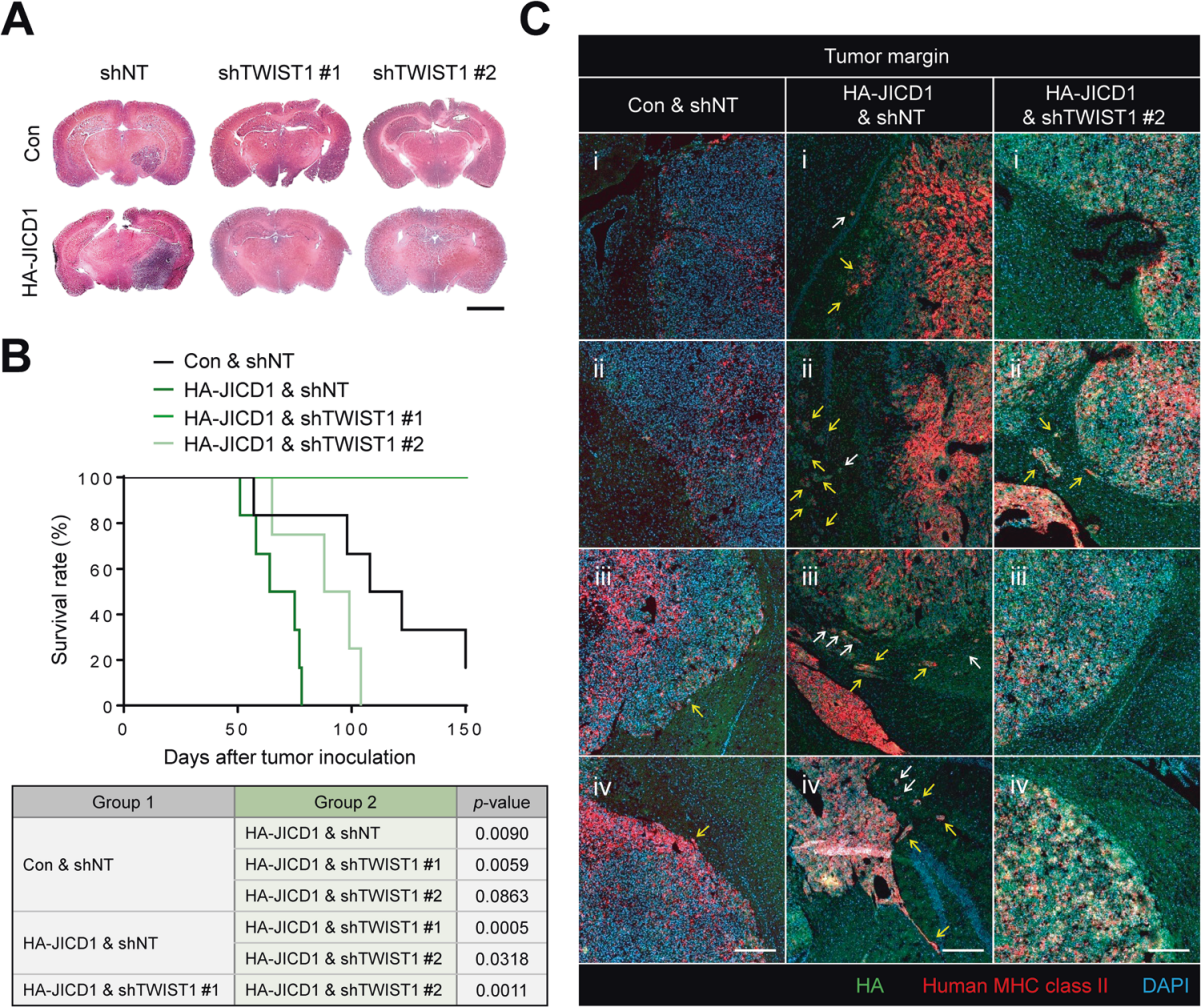
JICD1 promotes tumor invasion and aggressiveness via TWIST1 in vivo. **A** Representative images of hematoxylin-eosin staining of mouse brain sections 59 days after intracranial injections of U87MG control and HA-JICD1-overexpressing cells with TWIST1 knockdown. Scale bar = 2500 µm. **B** Survival graph of tumor-bearing mice after intracranial injections of U87MG control and HA-JICD1-overexpressing cells with TWIST1 knockdown. Statistical significance was tested by log-rank test. *p < 0.05, **p < 0.01, ***p < 0.001. **C** Images showing the invasive margin regions in mouse brain sections after intracranial injections of U87MG control and HA-JICD1-overexpressing cells with TWIST1 knockdown. White arrow indicates single cell migration and yellow arrow shows collective migration. Scale bar = 100 µm.

Given that the JICD1 transcriptional complex regulates cell migration and invasion via TWIST1, we investigated the functional consequences of JICD1/SMAD3-TWIST1-mediated cell migration on tumor aggressiveness in vivo. To this end, we orthotopically injected control and JICD1-overexpressing U87MG cells with or without TWIST1 knockdown and compared tumorigenicity on the same day (Fig. 5A). JICD1 overexpression results in increased tumorigenicity which is abolished under TWIST1 deficient conditions. As shown in a previous finding that inhibition of TWIST1 results in cell-cycle arrest and induction of cell death [46], TWIST1 knockdown also hindered tumorigenesis in control tumors (Fig. 5A).

Tumor aggressiveness is characterized by a faster growth rate, resistance to cell death, and the ability to penetrate surrounding tissues [47]. Proliferative phenotypes and invasiveness are segregated processes in malignancy. For example, hepatocyte growth factor induces cell proliferation and invasion separately via the Myc and mitogen-activated protein kinase (MAPK) signaling pathways, respectively [48]. Thus, to further understand tumor invasiveness specifically induced by JICD1/SMAD3-TWIST1 axis, we compared tumors derived from control, JICD1-overexpressing, and JICD1-overexpressing with TWIST1 knockdown U87MG cells. Control cells with TWIST1 knockdown were excluded because tumors derived from U87MG control cells had clear margins (Fig. 1E). As JICD1 promotes invasive phenotypes in vitro and in vivo (Figs 1, 2), JICD1 overexpression leads to poor prognosis in an intracranial xenograft model (Fig. 5B). TWIST1 knockdown JICD1-overexpressing cells prolonged the survival rate of mice, which corresponds to the finding that increased EMT markers and cell invasion were abolished in JICD1-overexpressing cells after TWIST1 knockdown (Figs 4D, E, 5B). Collectively, JICD1 promotes tumor aggressiveness through TWIST1 in vivo.

To analyze the histological characteristics of JICD1/SMAD3-TWIST1-activated tumors, tumors derived from control, JICD1-overexpressing, and JICD1-overexpressing cells transduced with short hairpin RNA (shRNA) #2 targeting TWIST1 were analyzed. Due to the difference in tumorigenicity following JICD1 overexpression and TWIST1 knockdown, the mice were sacrificed at the endpoint, and brain sections were obtained. shRNA #1 targeting TWIST1 was excluded, as it leads to extreme suppression of TWIST1 expression, which causes a rare population of migrating cells in 3D environment in vitro and no tumor formation in vivo (Figs 4D, E, 5A). JICD1 overexpression increased the expression of TWIST1 in vivo, in line with the finding that JICD1 transcriptionally activates TWIST1 in vitro (Fig. 2A, B, and Supplementary Fig. 14A). As TWIST1 diminished in JICD1-overexpressing cells after TWIST1 knockdown in vivo, the highly invasive characteristics induced by JICD1 were significantly repressed, corresponding to JICD1/SMAD3-TWIST1 axis induced cell invasion in vitro (Figs 4E, 5C and Supplementary Fig. 13). These results suggest that the invasive phenotype induced by JICD1/SMAD3-TWIST1 axis is a highly conserved pathway in gliomas in vitro and in vivo.

Furthermore, although a small population of cells was positive for Ki67, JICD1-overexpression increased the abundance of Ki67-positive population, which may cause increased tumorigenesis in JICD1-overexpressing tumors (Supplementary Fig. 14B). TWIST1 knockdown reversed the increase in the Ki67-positive population caused by JICD1. In contrast, the abundance of the cleaved caspase3-positive population was similar between the groups (Supplementary Fig. 14C). In vitro, cell proliferation and viability were not affected by JICD1 overexpression but decreased after TWIST1 knockdown (Supplementary Fig. 14D, E). Accordingly, the target genes of TWIST1 related to the cell cycle (*Trp53*, *Cdkn2a*, *Ccnd2*, *Cdk2* and *Cdk4*) and apoptosis (*Bax* and *Cdc25a*) [46, 49] showed similar mRNA expression levels in control and JICD1-overexpressing Ink4a/Arf^−/−^ astrocytes (Supplementary Fig. 14F). These results indicate that the JICD1/SMAD3-TWIST1 axis simultaneously regulates EMT and other pathways, promoting tumor malignancy.

Taken together, increased migration and invasion of JICD1-overexpressing cells is dependent on TWIST1, which is transcriptionally regulated by the JICD1 transcriptional complex. This phenomenon was evident both in vitro and in vivo.

### JICD1-TWIST1-MMP2 and MMP9 axes have clinical relevance in glioma patients

**Fig. 6:**
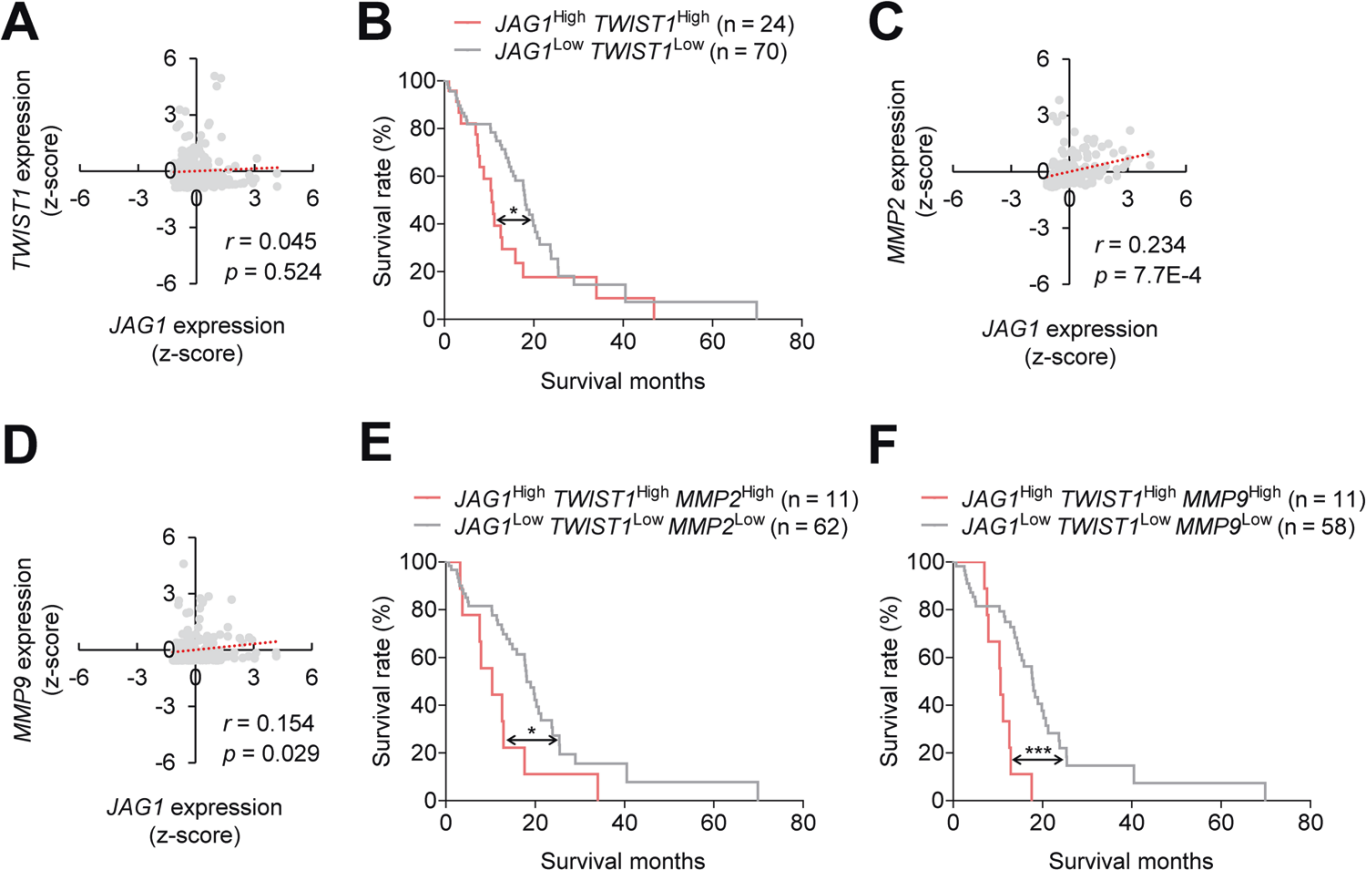
JICD1-TWIST1-MMP2 and MMP9 axes correlate with cancer aggressiveness in IDH wild-type GBM patients. **A** Correlation of JAG1 with TWIST1 in patients with IDH wild-type GBM using the TCGA GBMLGG database. **B** Survival rate of patients with IDH wild-type GBM according to the expressions of JAG1 and TWIST1. The patients were divided into two groups: JAG1-high and TWIST1-high vs JAG1-low and TWIST1-low based on mRNA expression (mean ± SEM). *P*-values were calculated by a log-rank (Mantel-Cox) test. *p < 0.05. **C**, **D** Correlation of JAG1 with JICD1/SMAD3-TWIST1 downstream signaling molecules (MMP2 and MMP9) in patients with IDH wild-type GBM using the TCGA GBMLGG database. **E**, **F** Survival rate of patients with IDH wild-type GBM according to the expressions of JAG1, TWIST1, MMP2, and MMP9. The patients were divided into two groups: JAG1-high, TWIST1-high, and MMP2-high vs JAG1-low, TWIST1-low, and MMP2-low (**E**) and JAG1-high, TWIST1-high, and MMP9-high vs JAG1-low, TWIST1-low, MMP9-low (**F**) based on mRNA expression (mean ± SEM). *P*-values were calculated by a log-rank (Mantel-Cox) test. *p < 0.05, ***p < 0.001.

Recent studies showed that more than 90% of primary GBM are isocitrate dehydrogenase (IDH) wild-type and IDH plays an important role in GBM aggressiveness [50]. Therefore, we analyzed the gene expression and prognosis of patients with IDH wild-type GBM to investigate the clinical relevance of JICD1/SMAD3-TWIST1 axis. Although JAG1 was weakly correlated with TWIST1, patients with high expression of both JAG1 and TWIST1 showed poor prognosis compared to those with low expression (Fig. 6A, B). As MMP2 and MMP9 are conserved downstream targets of the JICD1/SMAD3-TWIST1 axis (Figs 2A, 4D), we further analyzed the correlation between the mRNA expression of JAG1 and JICD1/SMAD3-TWIST1 downstream signaling molecules (MMP2 and MMP9) in IDH wild-type GBM patients (Fig. 6C, D). Both MMP2 and MMP9 were significantly associated with JAG1 expression. Moreover, prognostic analysis of patients with high or low expression of JICD1-TWIST1-MMP2 or JICD1-TWIST1-MMP9 demonstrated that both groups of genes were significantly correlated with prognosis (Fig. 6E, F). In contrast, neither JAG1-TWIST1-MMP2 nor JAG1-TWIST1-MMP9 axis was correlated with the prognosis of IDH-mutant glioma patients (Supplementary Fig. 15). Collectively, the JICD1/SMAD3-TWIST1-MMP2 and MMP9 axes have clinical relevance in IDH wild-type GBM patients specifically.

**Fig. 7:**
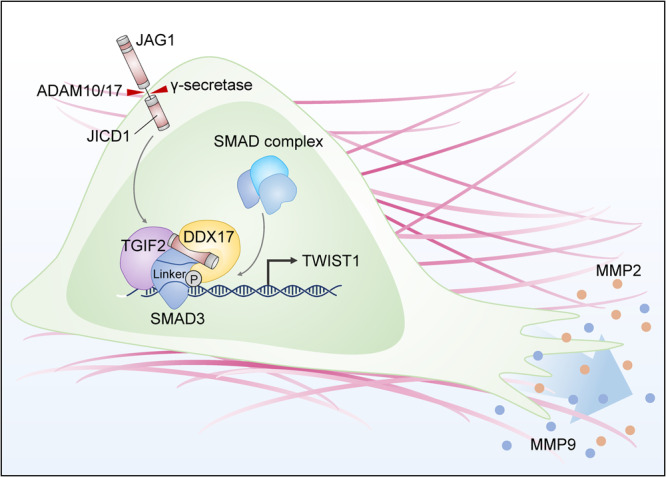
JICD1/SMAD3-TWIST1 axis induces tumor invasion. A graphic summary describing JICD1-induced cell migration and invasion. JICD1 is a transcription cofactor with inhibitory SMAD3. This complex promotes TWIST1 expression, which is crucial for canonical TGF-β signaling-independent cell migration and invasion.

In summary, our findings indicate that the JICD1 transcriptional complex contributes to the invasive phenotype of glioma cells with DDX17 and linker-phosphorylated SMAD3. This protein complex promotes cell migration and invasion through the transcriptional activation of TWIST1 (Fig. 7). The migratory and invasive phenotypes induced by the JICD1 transcriptional complex in GBM were distinct from TGF-β signaling. Taken together, the JICD1/SMAD3-TWIST1-MMP2 and MMP9 axes regulate the aggressive behavior of malignant gliomas. Therefore, the JICD1 transcriptional complex is a compelling therapeutic target for treating glioma malignancy.

## Discussion

Although JAG1 is mainly known as a ligand of Notch, the oncogenic function of JAG1 itself has recently been indicated [15, 51, 52]. Additionally, JICD1 formation induced by oncogenic signaling has been elucidated [14, 15]. JICD1 induces tumor progression; however, the molecular mechanisms by which JICD1 induces oncogenic phenotypes are unclear.

In the present study, we demonstrated the role of JAG1 in inducing aggressive behavior and invasion of the brain parenchyma. We also investigated the molecular mechanism of JICD1-induced tumor invasion in depth. JICD1 processed from JAG1 forms a transcriptional complex with linker-phosphorylated SMAD3 and transcriptionally regulates EMT. Specifically, the JICD1/SMAD3 complex promotes the transcriptional activation of TWIST1/MMP2 and MMP9 and regulates cell migration (Fig. 7). Additionally, the migratory phenotype induced by JICD1 was not associated with canonical TGF-β signaling which is a representative regulator of EMT.

Gliomas can originate from neural stem cells, oligodendrocyte progenitor cells, or differentiated glial cells [53]. These cells are driven by the neuroepithelial lineage and thus exhibit distinct molecular and behavioral properties compared with classical epithelial cells [54]. Unlike epithelial cells, canonical cadherin switching from E-cadherin to N-cadherin does not correlate with the mesenchymal phenotype of glioma cells [28]. Thus, “glial-to-mesenchymal transition” or EMT-like process has been proposed for malignant glioma [28]. Although the relevance of EMT in malignant gliomas remains controversial, malignant gliomas exhibit highly migratory and invasive characteristics. The expression of SNAIL, SLUG, TWIST, ZEB1, and ZEB2 enables glioma cells to shift toward a mesenchymal phenotype [28]. Consistent with these findings, our results indicated that JICD1 induces a migratory phenotype in glioma cells through the activation of TWIST1.

SMAD proteins are signaling transducers transmitting cellular signals to the nucleus and directly regulating downstream target transcription [40, 41]. In the canonical pathway, R-SMADs (SMAD1, SMAD2, SMAD3, SMAD5, and SMAD8) function as substrates of the TGF-β receptor family upon ligand binding [41, 55]. Specifically, the binding of TGF-β to its receptor phosphorylates SMAD2 and SMAD3, resulting in heterodimeric complex formations of two R-SMADs and one SMAD4 [41, 55]. Thus, the SMAD complex is a well-defined mediator of TGF-β-induced EMT [56]. Our findings showed that JICD1 forms a protein complex including SMAD3 and regulates the transcription of TWIST1. JICD1-overexpressing cells showed decreased levels of phosphorylated SMAD2. Nevertheless, JICD1-overexpressing cells showed increased expression levels of EMT-TF and markers after treatment with a TGF-β signaling inhibitor, SB431542 and LY2109761. These results indicate that JICD1 induces a migratory phenotype in glioma cells, distinct from canonical TGF-β signaling and SMAD activation. Additionally, while both inhibitors target ALK5, LY2109761 additionally inhibits TGF-β receptor II. LY2109761 decreased EMT-related gene expression unlike SB431542. In a previous study, JICD1 formation is regulated by various oncogenic signaling, such as KRAS/ERK/ADAM17 signaling, and induces EMT-related gene expression [15]. During EMT, various signaling pathways interact and share downstream effectors forming complex network [57]. However, the connection between TGF-β receptor II and those oncogenic signaling is not known at this stage. Thus, further investigation of TGF-β receptor II-regulated oncogenic signaling which enhances JICD1 formation is needed.

In the canonical TGF-β signaling pathway, linker domain phosphorylation of SMAD3 is followed by C-terminal phosphorylation and suppression of TGF-β-induced responses [58, 59]. Interestingly, a recent study revealed that inhibition of SMAD3 linker domain phosphorylation results in reduced tumor growth and metastasis [60]. Our results also indicated that phosphorylation of the SMAD3 linker domain may be crucial for the formation of the JICD1 transcriptional complex and glioma cell invasion. SMAD3 is phosphorylated in the linker domain by various proline-directed kinases, such as CDK2/4, JNK, ERK, MAPK, and GSK3β [61]. Thus, the upstream signaling pathway, which regulates the phosphorylation of the SMAD3 linker domain, is a key factor in JICD1 transcriptional complex formation. Additionally, there may be crosstalk between the JICD1 transcriptional complex and other signaling pathways.

Furthermore, inhibition of C-term phosphorylation of SMAD3 resulted in modest decrease in JICD1 transcriptional complex formation. Previous studies have demonstrated that C-term phosphorylation of R-SMADs induces conformational changes in proteins, resulting in protein complex formation and stable protein-protein interactions [43, 44]. In our results, C-terminal phosphorylation of SMAD3 was significantly upregulated in A172-HA-JICD1 cells than in control cells, but not in U87MG-HA-JICD1 cells. In addition, JICD1-overexpressing A172 cells display greater increase in the expression of EMT-TFs and markers compared to the influence in U87MG cells. Our results also suggest that C-terminal phosphorylation of SMAD3 regulates stability of the JICD1 transcriptional complex.

The JICD1 transcriptional complex upregulates the expression of TWIST1 and induces an aggressive GBM phenotype. TWIST1 is a transcription factor critical for mesodermal development during embryogenesis and is preferentially expressed in mesenchymal stem cells and mesoderm-derived tissues in adult humans, such as the placenta, skeletal muscle, and adipocytes [62–64]. For example, TWIST1 regulates the growth and lineage commitment in mesenchymal stem cells [63]. Additionally, inflammatory adipokine secretion and fatty acid oxidation in white adipocytes may be regulated by TWIST1 [64]. Thus, inhibition of EMT-TFs, including TWIST1, to prevent cancer invasion during treatment can affect normal cell physiology. As JAG1 is associated with tumorigenesis and aggressiveness in various tumors, targeting JAG1 may be a suitable strategy for anti-cancer therapy. Recently, monoclonal antibodies against JAG1 have been developed for clinical application [65]. After treatment with JAG1 antibodies, the target genes of Notch signaling were downregulated [65]. Thus, direct targeting of JAG1 may block Notch signaling, which is important for tissue homeostasis and causes unintended effects. Another strategy for inhibiting the oncogenic function of JAG1 is to prevent JICD1 formation by inhibiting γ-secretase. However, γ-secretase is also involved in the proteolysis of various proteins, including Notch, E-cadherin, and ErbB-4 [66–68]. Although numerous clinical trials of γ-secretase inhibitors have been conducted, several side effects have been reported due to the indiscriminateness of γ-secretase [69, 70]. Recently, aberrant protein-protein interactions (PPIs) have been proposed as the cause of various diseases, including cancer [71, 72]. Additionally, cancer-specific PPIs (oncoPPIs) have been identified [73]. JAG1 is upregulated in GBM than in non-tumor cells, and its expression correlates with tumor aggressiveness. In aggressive tumors, targeting the PPI of JICD1 transcriptional complex components to inhibit EMT-TFs would have therapeutic advantages [74].

In this study, we elucidated that the JICD1 transcriptional complex induces the migratory and invasive phenotypes. Because the role of JICD1 in inducing aggressive phenotype was verified in GBM cell lines, JICD1-driven tumor invasion needs to be explored in larger patient-derived tumorsphere cohorts. Moving forward, it remains to be studied in other types of human cancers, given that the JICD1-TWIST1-MMP2 and MMP9 axes correlate with GBM aggressiveness, and nuclear-localizing JAG1 is observed in glioma patients. Also, the upstream regulator of JICD1 and SMAD3 complex formation is unclear. Further investigation of various protein kinases likely to regulate SMAD3 linker domain phosphorylation and the formation of the JICD1 transcriptional complex is needed.

In addition to migratory and invasive phenotypes, the JICD1 transcriptional complex induces stemness phenotypes, including tumorigenesis and drug resistance in GBM [16]. Given that intratumoral heterogeneity is a major hurdle in cancer therapy, attempts have been made to therapeutically target the complexity of tumors through combination therapy [75]. Cancer cells with diverse characteristics among heterogeneous populations in tumors can be targeted through the inhibition of JICD1 transcriptional complex formation.

## Materials and methods

### Cell lines and culture conditions

The human glioma cell lines A1207 (RRID: CVCL_8481), A172 (RRID: CVCL_0131), LN18 (RRID: CVCL_0392), LN229 (RRID: CVCL_0393), T98G (RRID: CVCL_0556), and U87MG (RRID: CVCL_0022) were purchased from American Type Culture Collection (ATCC, Manassas, VA, USA). Human glioma cell lines were cultured in Dulbecco’s Modified Eagle’s Medium (DMEM, Cat. #17-605E; Lonza, Basel, Switzerland) supplemented with 10% fetal bovine serum (FBS, Cat. #7101; Biotechnics Research, Inc., Mission Viejo, CA, USA), 1% penicillin and streptomycin (P/S, Cat. #17-602E; Lonza), 1% L-glutamine (Cat. #17-605E; Lonza), and gentamicin sulfate (50 μg/mL, Cat. #6442; Tocris Bioscience, Bristol, UK). The human glioma stem cell line GSC20 was established from human brain tumor [76]. GSC20 was cultured in DMEM and Ham’s F-12 (Cat. #SH30023.01; Cytiva, Marlborough, MS, USA) supplemented with 1% P/S, 1% L-glutamine, 0.2% B27, epidermal growth factor (20 ng/ml, Cat. #236-EG-01M; R&D systems, Minneapolis, MN, USA), basic fibroblast growth factor (20 ng/mL, Cat. #4114-TG; R&D systems), and gentamicin sulfate (50 μg/mL). Normal human astrocytes were purchased from ScienCell (Cat. #1800-5; Carlsbad, CA, USA) and maintained in Astrocyte Medium (Cat. #1801; ScienCell) supplemented with 2% FBS (Cat. #0010; ScienCell), 1% Astrocyte Growth Supplement (AGS, Cat. #1852; ScienCell) and 1% P/S (Cat. #0503; ScienCell). All human cell lines were authenticated using Short Tandem Repeat profiling. Conditions that inhibited canonical TGF-β signaling were induced using an ALK5 (TGFBR) inhibitor (10 μM for A172 and 20 μM for U87MG) (SB431542, Cat. #S1067; Selleckchem, Houston, TX, USA) and ALK5/TGFBRII dual inhibitor (5 μM for A172 and U87MG) (LY2109761, Cat. #15409; Cayman Chemical, Ann Arbor, MI, USA). The endogenous JICD1 formation was inhibited by 10 μM γ -secretase inhibitor (DAPT, Cat. #D5942; Sigma-Aldrich, Burlington, MA, USA).

### Vector construction and lentivirus infection

Human HA-JICD1 was cloned into pLL-CMV-blast, and HA-DDX17 and FLAG-SMAD3 were cloned into pcDNA3.1-puro vector. SMAD3 mutants were established using an EZchange^TM^ site-directed mutagenesis kit (Cat. #EZ004S; Enzynomics, Daejeon, Republic of Korea) and cloned into the pcDNA3.1-puro vector. Short hairpin RNAs targeting TWIST1 were cloned into pLKO.1-puro.

To construct HA-JICD1 overexpressing A172 and U87MG cells, pLL-CMV-HA-JICD1-blast vector was transfected with second-generation lentiviral packaging plasmids Δ8.9 and pVSV.G using the PolyExpress^TM^ in vitro DNA transfection reagent (Cat. #EG1072; Excellgen, Rockville, MD, USA) in transformed HEK293T cells (Cat. #CRK-3216; ATCC). The culture medium was harvested 24 h after transfection. The lentivirus was filtered through a 0.45 μm syringe filter and concentrated with a Lenti-X^TM^ Concentrator (Cat. #631231; Clontech, Mountain View, CA, USA). A172 and U87MG cells were infected with the virus in the presence of 6 μg/mL polybrene (Cat. #107689; Sigma-Aldrich). Stable A172-HA-JICD1 and U87MG-HA-JICD1 cells were selected in DMEM supplemented with 10% FBS and 1 μg/mL blasticidin (Cat. #46-1120; Invitrogen, Waltham, MA, USA) for seven days.

For TWIST1 knockdown, pLKO.1-puro vectors with shRNA targeting TWIST1 were transfected into HEK293T cells to produce lentivirus. Control and JICD1-overexpressing A172 and U87MG cells were infected with the virus. The target mRNA sequences are as follow: shTWIST1 #1-GCATTCTGATAGAAGTCTGAA, shTWIST1 #2-GGAACTATAAGAACACCTTTA.

To confirm the interaction between the proteins, the HA-DDX17, FLAG-SMAD3, and FLAG-SMAD3 mutant vectors were transfected using the PolyExpress^TM^ in vitro DNA transfection reagent into HEK293T cells. Thirty-six hours after transfection, cells were treated with 5 μM MG-132 (Cat. #M7449; Sigma-Aldrich) for 12 h and harvested to verify the protein expression and interaction.

### Quantitative reverse transcription-polymerase chain reaction (qRT-PCR)

Total RNA was isolated from cells using the QIAzol lysis reagent (Cat. #79306; Qiagen, Valencia, CA, USA) according to the manufacturer’s instructions. RNase-free DNase-treated RNA (1 μg) was used as a template to synthesize cDNA using the RevertAid First-Strand cDNA Synthesis Kit (Cat. #K1621; ThermoFisher Scientific, Waltham, MA, USA). qRT-PCR analysis was performed on an iCycler IQ real-time detection system (Bio-Rad, Hercules, CA, USA) using IQ Supermix with SYBR Green (Cat. #RR420A; TaKaRa Bio, San Jose, CA, USA). Gene expression was quantified using the standard 2 − ∆∆Ct method as previously described. The expression levels of target genes were normalized to those of 18 S rRNA. The primers for qRT-PCR amplification used were as follows: 18 S rRNA (forward) TGCATGGCCGTTCTTAGTTG/(reverse) AGTTAGCATGCCAGAGTCTCGTT, TWIST1 (forward) CCACTGAAAGGAAAGGCATC/(reverse) TGCATTTTACCATGGGTCCT, SNAI1 (forward) CCCAATCGGAAGCCTAACTA/(reverse) GGCTGCTGGAAGGTAAACTC, SNAI2 (forward) TGGTTGCTTCAAGGACACAT/(reverse) GCAGATGAGCCCTCAGATTT, ZEB1 (forward) TGCACTGAGTGTGGAAAAGC/(reverse) TGGTGATGCTGAAAGAGACG, ZEB2 (forward) ACGGTATTGCCAACCCTCTG/(reverse) GGTCTGGATCGTGGCTTCTG, CDH1 (forward) CATCTCCCTTCACAGCAGAA/(reverse) CTAAGGCCATCTTTGGCTTC, CDH2 (forward) CTTGCCAGAAAACTCCAGGG/(reverse) TGTGCCCTCAAATGAAACCG, FN1 (forward) GGCCAGTCCTACAACCAGTA/(reverse) CTCTCGGGAATCTTCTCTGTC, ACTA2 (forward) AATGGCTCTGGGCTCTGTAA/(reverse) TCTTTTGCTCTGTGCTTCGTC, VIM (forward) TCA AGAACACCCGCACCAA/(reverse) CGGGCTTTGTCGTTGGTTAG, IL6 (forward) GAGTAGTGAGGAACAAGCCAGAG/(reverse) GCATTTGTGGTTGGGTCAGGG, IL8 (forward) CCTAGATATTGCACGGGAGA/(reverse) GCTTCCACATGTCCTCACAA, MMP2 (forward) TTGACGGTAAGGACGGACTC/(reverse) CTCCCAAGGTCCATAGCTCA, MMP9 (forward) GCACCACCACAACATCACCTA/(reverse) GACACCAAACTGGATGACGATG, SMAD2 (forward) CCGGAGAGTCAAGTTTCTGC/(reverse) AAGTGACCCTGCCTCAGCTA, SMAD3 (forward) CCCCAGAGCAATATTCCAGA/(reverse) GGCTCGCAGTAGGTAACTGG, SMAD4 (forward) GGGACCGGATTACCCAAGAC/(reverse) GCCCCAACGGTAAAAGACCT, SMAD6 (forward) TGAATTCTCAGACGCCAGCA/(reverse) AGTACGCCACGCTGCACC, SMAD7 (forward) AGCAGGCCACACTTCAAACT/(reverse) GTGTCCTGCCGATCATACCT, TGFB1 (forward) TTTGATGTCACCGGAGTTGT/(reverse) TGCAGTGTGTTATCCCTGCT, TGFB2 (forward) GCTAAAATTCTTGGAAAAGTGGC/(reverse) TTTTAACACTGATGAACCAAGGC, TGFB3 (forward) GGGTTTGGTTAGAGGAAGGC/(reverse) CCATTGCCACACAACATCTC, TGFBR1 (forward) AACTTCCAACTACTGGCCCTT/(reverse) GGTGAATGACAGTGCGGTTG, TGFBR2 (forward) GGGGAAACAATACTGGCTGA/(reverse) TCACACAGGCAGCAGGTTAG, TGFBR3 (forward) CTTTCCTCTTCCCAGCGAGT/(reverse) TGTGTCCAGCGGAGATCCA.

### Co-IP and western blot analysis

For the Co-IP experiment, HEK293T cells co-transfected with the previously described plasmids or stable HA-JICD1 overexpressing A172 and U87MG cells were used. The proteins in these cells were extracted using Pierce^TM^ IP lysis buffer (Cat. #87787; Invitrogen) supplemented with 1 mM phenylmethylsulfonyl fluoride (PMSF, Cat. #10837091001; Sigma-Aldrich), protease inhibitors (Cat. #11836-153-001; Roche, Basel, Switzerland) and phosphatase inhibitors (1 mM NaF, 1 mM Na_3_VO_4_, 1 mM β-glycerophosphate, and 2.5 mM sodium pyrophosphate). Lysates were pre-cleared with Protein A/G agarose (Cat. #20333 and #20399; Thermo Fisher Scientific), and the proteins (0.5–1 mg) were precipitated using anti-HA antibody (1:100, Cat. #3724 s; Cell Signaling Technology, Danvers, MA, USA), IgG isotype control (1:100, Cat. #10500 C; Thermo Fisher Scientific), and Protein A/G agarose. HA-binding proteins were washed with IP buffer and eluted with NuPAGE^TM^ LDS sample buffer (Cat. #NP0007; Thermo Fisher Scientific) for 3‒5 min at 100 °C. The eluted proteins were separated using SDS-polyacrylamide gel electrophoresis (PAGE) and transferred to polyvinylidene fluoride membranes (Cat. #BSP0161; Pall Corporation, Tokyo, Japan). The membranes were blocked with 5% non-fat milk and incubated with the following antibodies: anti-FLAG (1:1000, Cat. #F1804; Sigma-Aldrich), anti-HA (1:1000, Cat. #H9658; Sigma-Aldrich), anti-DDX17 (1:1000, Cat. #sc-271112; Santa Cruz Biotechnology, Dallas, TX, USA), anti-pSMAD3 (Ser 423/425; 1:1000, Cat. #ab52903; Abcam, Cambridge, UK), anti-pSMAD3 (Ser 213; 1:1000, Cat. #ab63403; Abcam), and anti-SMAD3 (1:1000, Cat. #9523; Cell Signaling Technology). After washing, the membranes were incubated with a horseradish peroxidase-conjugated anti-IgG secondary antibody (Pierce, Waltham, MA, USA) and visualized using SuperSignal West Pico Chemiluminescent Substrate (Cat. #EBP-1073; ELPIS Biotech, Daejeon, Republic of Korea).

For western blotting, whole-cell extracts were prepared using radioimmunoprecipitation assay (RIPA) lysis buffer (Cat. #CBR002; LPS solution, Daejeon, Republic of Korea) comprising 150 mM NaCl, 1% NP-40, 0.1% SDS, and 50 mM Tris (pH 7.4) containing 1 mM NaF, 1 mM Na_3_VO_4_, 1 mM β-glycerophosphate, 2.5 mM sodium pyrophosphate, and protease inhibitor (Cat. #11836-153-001; Roche). Proteins were quantified using the Bradford assay reagent (Cat. #500-0006; Bio-Rad), according to the manufacturer’s instructions. Protein (10–50 μg) was separated by 8–12% SDS-PAGE and immunoblotting was performed as described above. The primary antibodies were used with the following antibodies: anti-HA (1:1000, Cat. #3724s; Cell Signaling Technology), anti-VIM (1:1000, Cat. #ab16700; Abcam), anti-α-SMA (1:5000, Cat. #A2547; Sigma-Aldrich), anti-ZEB2 (1:1000, Cat. #sc-271984, Santa Cruz Biotechnology), anti-TWIST1 (1:1000, Cat. #MA5-17195, Invitrogen), anti-SLUG (1:500, Cat. #9585; Cell Signaling Technology), anti-pSMAD3 (Ser 423/425) (1:1000, Cat. #ab52903; Abcam), anti-pSMAD3 (Ser 213) (1:1000, Cat. #ab63403; Abcam), anti-SMAD3 (1:1000, Cat. #9523; Cell Signaling Technology), anti-pSMAD2 (Ser 465/467) (1:1000, Cat. #3108; Cell Signaling Technology), anti-SMAD2 (1:1000, Cat. #5339; Cell Signaling Technology), anti-JAG1(1:1000, Cat. #70109 s; Cell Signaling Technology), anti-β-actin (1:10000, Cat. #sc-47778; Santa Cruz Biotechnology), and anti-α-tubulin (1:10000, Cat. #T6199; Sigma-Aldrich). β-actin and α-tubulin were used as loading controls.

### Cell viability assay and cell confluency quantification

Cell viability was evaluated using a Cyto X cytotoxicity assay kit (Cat. #CYT3000; LPS Solution). First, 1 × 10^3^ cells (A172 and U87MG) were seeded in each well of 96-well plates. Cyto X solution was added to each well on days 0, 1, 3, and 5 after seeding, followed by incubation for 2 h. Absorbance was recorded at 450 nm using a Multi-Detection Microplate Reader (Cat. #Sense 425-301; Hidex, Levi, Finland). The relative cell viability was normalized to the absorbance on day 0 for each cell line.

To analyze changes in cell confluency, 1 × 10^3^ cells (A172 and U87MG) were seeded in each well of 96-well plates. Each well was scanned at 4-h intervals using IncuCyte (IncuCyte Zoom; Sartorius, Niedersachsen, Germany), and the relative cell confluency was normalized to that of day 0 for each cell line.

### Wound closure assay

For the wound closure assay, 4 × 10^5^ cells (A172 and U87MG) were seeded in six-well plates at 100% confluence for 24 h. After 24 h, the cells were incubated with mitomycin C (10 μg/mL) for 1 h. An incision was made in the central area of the confluent culture plate to create an artificial wound. Images of the wound area were captured using a microscope until the wound closure, and the area was quantified using ImageJ software (https://imagej.nih.gov).

### Matrigel invasion assay

Invasion assays were performed using 24-well transwell units (Cat. #3422; Corning Coster, Glendale, AZ, USA) coated with 1 mg/mL Matrigel (1:100, Cat. #354277, Corning Coster), which was allowed to settle for 4 h at 37 °C. Next, 5 × 10^4^ cells (A172 and U87MG) suspended in 100 μL serum-free DMEM were seeded into the upper chamber, and 500 μL of serum-containing DMEM was added to the lower chamber. Cell invasion occurred for 48 h at 37°C. Migration assays were performed using 24-well transwell units without Matrigel coating, wherein 3 × 10^3^ cells of A172 or 1 × 10^4^ cells of U87MG suspended in 200 μL of serum-free DMEM were seeded into the upper chamber, and 500 μL of serum-containing DMEM was added to the lower chamber. Cell migration occurred for 48 h at 37 °C.

Next, non-migrated cells from the upper chamber were removed, and migrated cells on the lower side of the membrane were fixed, stained with crystal violet, and dried. Images of the entire membrane of upper chamber were obtained, and the invasion area was quantified using ImageJ software.

### 3D microchip cell invasion assay

Before cell seeding, pre-polymerized type I collagen solution (3.25 mg/mL in 10× phosphate-buffered saline [PBS] with phenol red at pH 7.4) was introduced into the gel inlet of the AIM 3D Cell Culture Chip (Cat. #DAX-1; AIM Biotech, Central Region, Singapore) and placed in the humidified 5% CO_2_ incubator at 37 °C for 45 min to polymerize the solution. After incubation, media channels were filled with DMEM or fibronectin coating solution (diluted to 25 μg/mL fibronectin in DMEM) and microchips plated in the humidified incubator at 37 °C for 1 h. Next, 3 × 10^4^ cells (A172 and U87MG) were seeded into fibronectin coated channels of microchip maintained in the humidified 5% CO_2_ incubator at 37 °C for 1 h in a tilted position to facilitate attachment of cells by gravity. DMEM without supplements was introduced in the cell culture channels and DMEM containing 10% FBS, 1% P/S, 1% L-glutamine was filled in opposite channel. Media in all channels were exchanged with fresh media every 12 h to maintain the linear concentration gradient until the cells had sufficiently migrated. Finally, the cells were stained overnight at 4 °C with following antibodies; anti-HA (1:200, Cat. #3724s; Cell Signaling Technology), anti-α-SMA (1:200, Cat. #A2547; Sigma-Aldrich), and anti-TWIST1 (1:200, Cat. #MA5-17195, Invitrogen). Then, the samples were washed in 3% BSA in PBS and incubated with Alexa 568 conjugated phalloidin (1:400, Cat. #A12380; Invitrogen), Alexa 488 conjugated secondary antibody (1:400, Cat. #A28175; Invitrogen), and Alexa 647 conjugated secondary antibody (1:400, Cat. #A11012; Invitrogen) according to the manufacturer’s instructions. After staining with 4’,6-diamidino-2-phenylindole (DAPI, 1:1000, Cat. #D9542; Sigma-Aldrich), fluorescence was detected using a confocal laser-scanning microscope (LSM800 and LSM900; Carl Zeiss, Oberkochen, Germany). Images were quantified using ImageJ.

### Immunofluorescence (IF)

To investigate endogenous JAG1 expression in the intracranial xenograft tumor model, tissue slides were permeabilized with PBS supplemented with 3% Triton X-100 and blocked with 3% BSA in PBS for 1 h at room temperature. The samples were stained with JAG1 antibody (1:200, Cat. #70109; Cell Signaling Technology) overnight at 4 °C, washed three times for 5 min each in ice-cold PBS, and incubated with Alexa 488 conjugated secondary antibody (1:400, Cat. #A32731, and #A28175; Invitrogen) or Alexa 594 conjugated secondary antibody (1:400, Cat. #A11012 and #11032; Invitrogen) at 25 °C for 2 h. After staining with DAPI (1:1,000, Cat. #D9542; Sigma-Aldrich) for 5 min, slides were mounted in mounting solution (Cat. #P36930; Invitrogen) and stored at 4 °C in the dark. Fluorescence was detected using a confocal laser-scanning microscope (LSM800; Carl Zeiss). Images were quantified using ImageJ.

To investigate invasive characteristics in tumor tissues, samples removed at the time of death were used (day 58 for control, 78 for HA-JICD1, and 89 for HA-JICD1-shTWIST1#2). Samples were stained with APC-conjugated human-specific HLA-DR, DP, and DQ antibodies (1:200, Cat. #130-123-843, Miltenyi Biotec, Gaithersburg, MD, USA) overnight at 4 °C. The samples were then washed and stained with HA antibody (1:200, Cat. #3724s; Cell Signaling Technology) and TWIST1 antibody (1:200, Cat. #MA5-17195, Invitrogen) overnight at 4 °C. The samples were again washed and incubated with Alex 488 and Alex 594 conjugated secondary antibodies for 2 h. After staining with DAPI, the slides were mounted. Fluorescence was detected using a confocal laser-scanning microscope (LSM800 and LSM900; Carl Zeiss).

To verify the histological characteristics of the tumor tissue, the samples were stained with Ki67 antibody (1:200, Cat. #NCL-Ki67; Leica Biosystems, Wetzlar, Germany) or cleaved caspase3 antibody (1:200, Cat. #9661, Cell Signaling Technology) overnight at 4 °C. The samples were then washed and incubated with Alexa 488 conjugated secondary antibody for 2 h. After staining with DAPI, the slides were mounted. Fluorescence was detected using a confocal laser-scanning microscope (LSM800; Carl Zeiss). Images were quantified using ImageJ.

### Intracranial xenograft model

LN229, U87MG, U87MG-control, or U87MG-HA-JICD1 cells transduced with shTWIST1 (*n* = 6) were harvested and washed with PBS. Cell viability was determined using the trypan blue exclusion method. Single-cell suspensions with >90% viability were used for in vivo experiments. The cells (1 × 10^5^ cells/3 μL PBS) were stereotactically injected into the left striatum of 5-week-old BALB/c nu/nu mice (coordinates relative to the bregma: medial-lateral +2 mm and dorsal-ventral -3 mm). The mice were randomly allocated to experimental groups and blinding was not done. To compare tumor histology, all mice were sacrificed simultaneously when the second mouse showed neurological symptoms. To compare the invasive phenotype after TWIST1 knockdown, brains were removed at the time of death. The Kaplan-Meier survival was determined within 150 days.

### Intracranial xenograft frozen section

Tumor-bearing mice were perfused with PBS and 4% paraformaldehyde (PFA). The tumor tissues were fixed in 4% PFA overnight at 4 °C and incubated in 30% sucrose in PBS for 72 h. The samples were then mounted in FSC 22 Frozen Section Media (Cat. #3801480, Leica Biosystems) and frozen at −20 °C. Frozen tissues were cut into 10 μm thick slices using a cryostat (CM1950; Leica Biosystems). The frozen sections were air dried for 30 min at room temperature and stored at −80 °C.

### Hematoxylin-eosin staining

To compare tumorigenicity, samples were dipped in hematoxylin (Cat. #1.05174.0500, Merck, Rahway, NJ, USA) 10 times and rinsed with tap water; they were treated with eosin (Cat. #1.09844.1000, Merck) for 6 min, followed by dehydration in xylene. Finally, the slides were mounted using mounting solution (Cat. #SP15-100; Thermo Fisher Scientific).

### ChIP assay

For crosslinking, control and HA-JICD1 overexpressing A172 and U87MG cells were treated with formaldehyde (final concentration: 1%) in the culture media for 10 min at 20 °C. The reaction was quenched with glycine (final concentration: 0.125 M) for 5 min at 20 °C. The cells were then washed twice with cold PBS and harvested using a cell scraper. The cell pellet was resuspended in lysis buffer (0.5% NP40, 85 mM KCl, 5 mM Pipes, 1 mM PMSF, 0.01 mg/mL aprotinin, and 0.01 mg/mL leupeptin). After incubation for 10 min on ice, the supernatant was removed by centrifugation (5000 rpm, 5 min), and the pellet was lysed with nuclear lysis buffer (0.5% SDS, 10 mM EDTA, 50 mM Tris pH 8.1, 1 mM PMSF, 0.01 mg/mL aprotinin, and 0.01 mg/mL leupeptin) for 10 min on ice. Cross-linked chromatin was fragmented to the 0.3–1.5 kb range by focused-ultrasonication. The insoluble fraction was removed by centrifugation and the supernatant was pre-cleared with Protein A/G/sperm DNA beads for 1 h at 4 °C. For ChIP, 300 µg chromatin DNA was incubated with anti-HA (Cat. #3724s; Cell Signaling Technology) and anti-SMAD3 (Cat. #9523; Cell Signaling Technology) at 4 °C overnight, followed by 50 µL of Protein A/G/sperm DNA beads for 2 h at 4 °C. The beads were washed and eluted. Chromatin crosslinking was reversed by adding 5 M NaCl at 65 °C overnight. Residual RNA was digested with RNase A for 30 min at 37 °C, and the DNA was purified using a DNA purification kit (Cat. #EBP-1004; ELPIS Biotech). Quantification of promoter DNA was performed using qRT-PCR with specific primers (Human TWIST1 promoter −1938 ~ −1795: forward-GTAGCGGAAGATGCAAACGC, reverse-ACTGGCCTGTTTAGTGAGCC). The relative amount of promoter DNA was normalized to that of the input.

### In silico analysis

To investigate the gene ontology enriched in JAG1-overexpressing cells, RNA-sequencing data of control and JAG1-overexpressing Ink4a/Arf^-/-^ astrocytes (GSE225528) or control and JICD1-overexpressing Ink4a/Arf^−/−^ astrocytes (GSE126855) were analyzed. ClueGO, a Cytoscape plugin for the visualization of functionally organized gene ontology/pathway networks, was used to determine the enriched biological terms [77].

To confirm the expression of EMT-TFs and the inhibition of TGF-β signaling by JICD1, RNA-sequencing data of control and JICD1-overexpressing Ink4a/Arf^−/−^ astrocytes (GSE126855) were analyzed. Further, Gene Set Enrichment Analysis was performed using TGF-β signaling gene sets derived from oncogenic signatures and MSigDB Hallmark (https://www.broadinstitute.org/gsea) [78, 79].

The mRNA expression of patients from the TCGA GBMLGG database generated by the TCGA Research Network (https://www.cancer.gov/tcga) was used to verify the clinical relevance of JICD1/SMAD3-TWIST1-MMP2 and MMP9 axes. Patients with IDH wild-type glioma with *TERT* gene promoter mutation or chromosome 7 amplification/loss of chromosome 10 were classified as IDH wild-type GBM patients. IDH mutation status was used for analyzing IDH-mutant glioma cohorts. The high- and low- expression groups were separated based on mRNA expression levels (mean ± standard error [SEM]).

### Quantification and statistical analysis

All experiments were replicated more than three times. Data were analyzed using two-tailed Student’s *t*-test and are reported as the mean ± SEM. For statistical analyses of the patient dataset, a log-rank (Mantel–Cox) test and two-tailed unpaired *t*-test were used and analyzed using GraphPad Prism 6. Statistical significance was based on *p* values. *p < 0.05; **p < 0.01; and ***p < 0.001 were considered statistically significant.

### Supplementary information

Supplementary figure

Original Data File

## Data Availability

The RNA sequencing data of Ink4a/Arf^−/−^ astrocytes transfected with pcDNA3.1-puro or pcDNA3.1-JAG1-puro generated in this study are publicly available from the Gene Expression Omnibus (GEO) dataset GSE225528. The RNA sequencing data of control and HA-JICD1 overexpressed Ink4a/Arf^−/−^ astrocytes analyzed in this study were obtained from GEO (GSE126855).
