# Supplementary material for: Jagged1 intracellular domain/SMAD3 complex transcriptionally regulates TWIST1 to drive glioma invasion
Source: Cell Death Dis. 2023 Dec 13;14(12):822. doi: 10.1038/s41419-023-06356-0 (PMC10719344; doi:10.1038/s41419-023-06356-0)
Supplement: Supplementary file 1 — Supplementary figure [file 41419_2023_6356_MOESM1_ESM.pdf]

Jagged1 intracellular domain/SMAD3 complex transcriptionally regulates TWIST1 to drive glioma invasion

Jung Yun Kim[1,2†], Nayoung Hong[1,2†], Sehyeon Park[1,2], Seok Won Ham[3], Eun-Jung Kim[3], Sung-Ok Kim[4], Junseok Jang[1,2], Yoonji Kim[1,2], Jun-Kyum Kim[3], Sung-Chan Kim[4], Jong-Whi Park[*5], and Hyunggee Kim[*1,2]

[1]Department of Biotechnology, College of Life Sciences and Biotechnology, Korea University, Seoul 02841, Republic of Korea

[2]Institute of Animal Molecular Biotechnology, Korea University, Seoul 02841, Republic of Korea

[3]MEDIFIC Inc., Hwaseong-si, Gyeonggi-do 18469, Republic of Korea

[4]Department of Biochemistry, College of Medicine, Hallym University, Chuncheon 24252, Republic of Korea

[5]Department of Life Sciences, Gachon University, Incheon 21999, Republic of Korea

[†]These authors contributed equally to this work.

[*]Correspondence: Hyunggee Kim (e-mail) hg-kim@korea.ac.kr

(Telephone) +82-02-3290-3059

Jong-Whi Park (e-mail) jpark@gachon.ac.kr

(Telephone) +82-032-899-6115

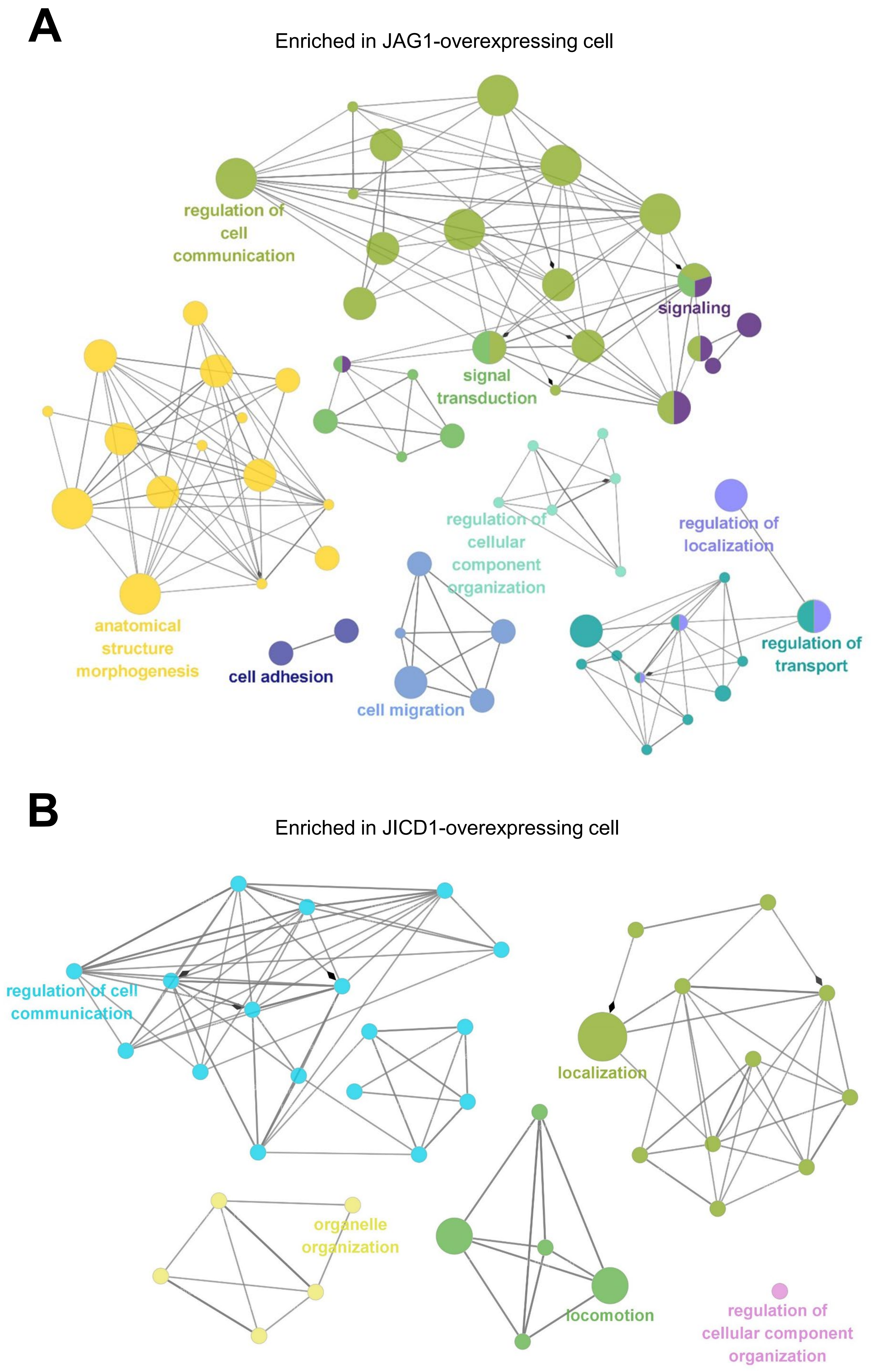

Supplementary Fig. S1 Gene ontology analysis on RNA-seq of JAG1- or JICD1-overexpressing Ink4a/Arf$^{-/-}$ astrocytes, Related to Fig. 1

ClueGo results demonstrated that biological processes were enriched in JAG1 (A) or JICD1 (B)-overexpressing Ink4a/Arf$^{-/-}$ astrocytes than in control cells.

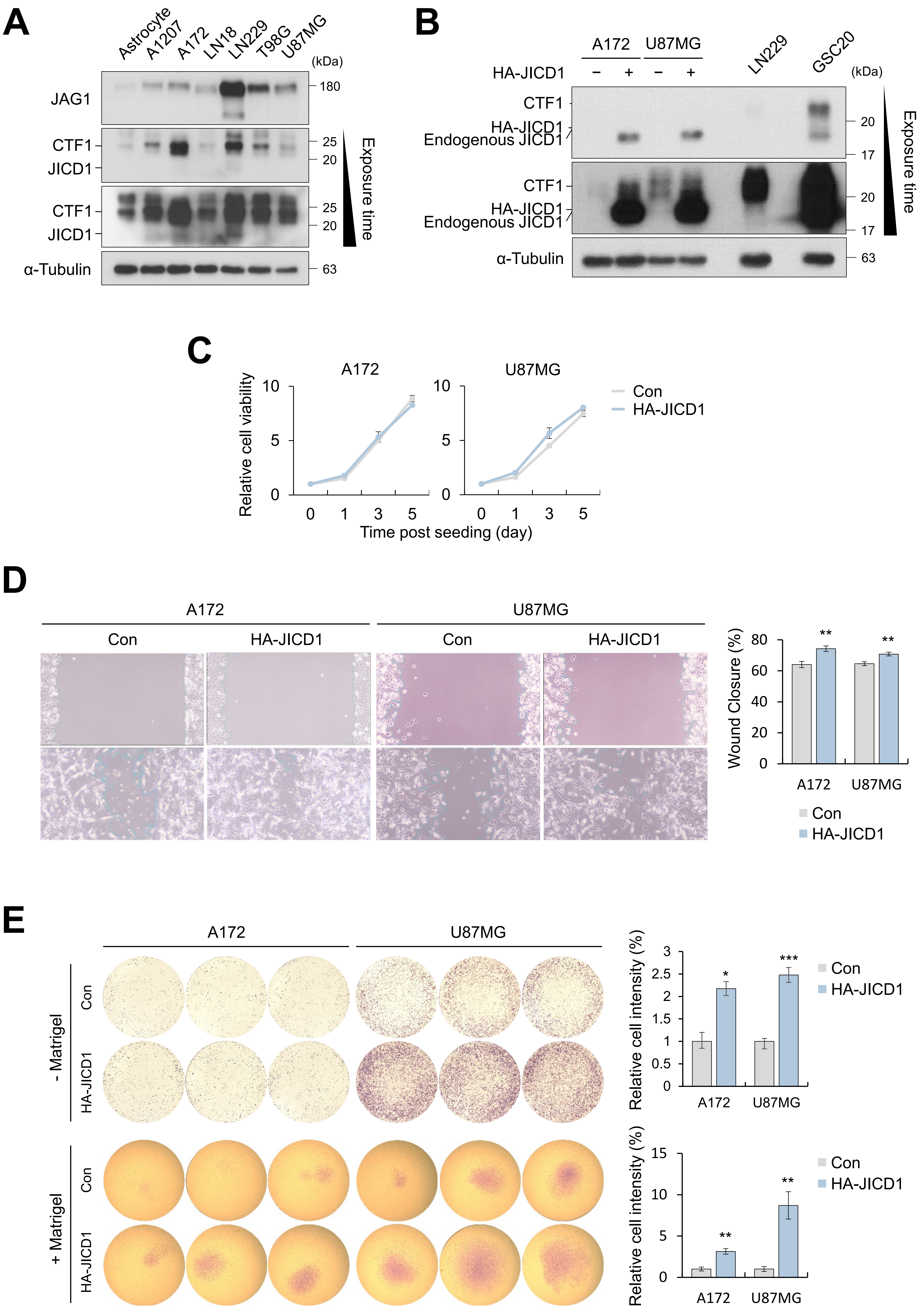
A
Astrocyte
A1207
A172
LN18
LN229
T98G
U87MG
(kDa)
JAG1
180
CTF1
JICD1
25
20
CTF1
JICD1
25
20
Exposure time
α-Tubulin
63
B
A172
U87MG
LN229
GSC20
HA-JICD1
−
+
−
+
(kDa)
CTF1
HA-JICD1
Endogenous JICD1
20
17
CTF1
HA-JICD1
Endogenous JICD1
20
17
Exposure time
α-Tubulin
63
C
A172
U87MG
Relative cell viability
10
5
0
0 1 3 5
Time post seeding (day)
Con
HA-JICD1
D
A172
U87MG
Con
HA-JICD1
Con
HA-JICD1
Wound Closure (%)
80
60
40
20
0
**
**
A172
U87MG
Con
HA-JICD1
E
A172
U87MG
- Matrigel
Con
HA-JICD1
+ Matrigel
Con
HA-JICD1
Relative cell intensity (%)
3
2.5
2
1.5
1
0.5
0
*
***
A172
U87MG
Con
HA-JICD1
Relative cell intensity (%)
15
10
5
0
**
**
A172
U87MG
Con
HA-JICD1

Supplementary Fig. S2 JAG1-derived JICD1 protein expression in GBM cell lines and migratory phenotype induced by JICD1, Related to Fig. 1

(A) Protein level of JAG1, CTF, and JICD1 in astrocytes and GBM cell lines (A1207, A172, LN18, LN229, T98G, and U87MG).

(B) Protein level of CTF, HA-JICD1, and endogenous JICD1 in A172 and U87MG control and HA-JICD1-overexpressing cells, LN229, and GSC20.

(C) Relative cell viability of A172 and U87MG control and HA-JICD1 overexpressing cells.

(D) Microscopic images showing the results of wound closure in A172 and U87MG control and HA-JICD1-overexpressing cells. A bar graph representing the average value of wound closure rates in eight different regions in the wound closure assay. $^{**}p < 0.01$.

(E) Microscopic images showing the results of the Matrigel invasion assays and transwell migration assays in A172 and U87MG control and HA-JICD1-overexpressing cells. A bar graph showing the average value of relative cell intensity in three transwells of the Matrigel invasion assay and transwell migration assay. $^{*}p < 0.05$, $^{**}p < 0.01$, $^{***}p < 0.001$.

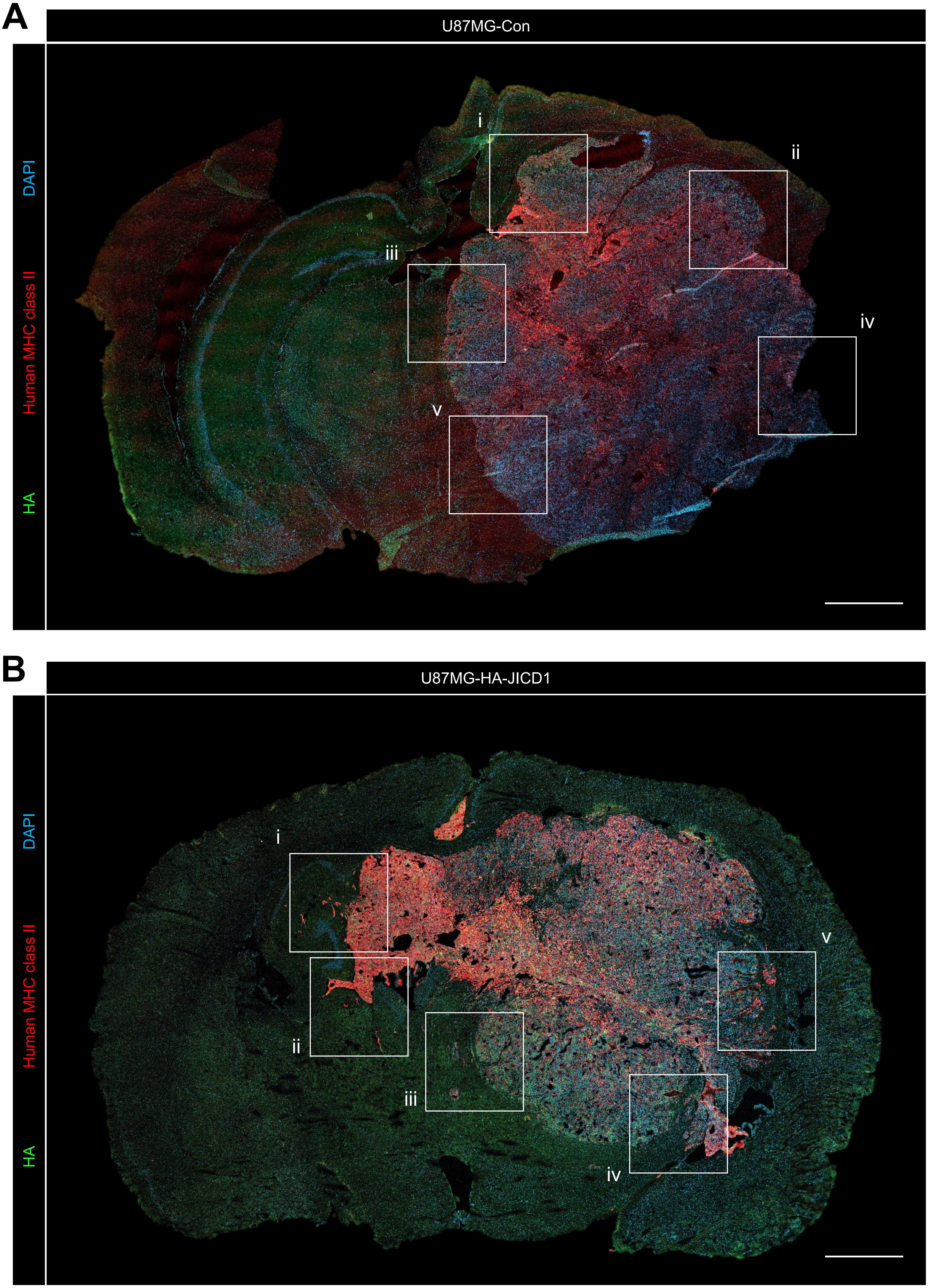
A
U87MG-Con
DAPI
Human MHC class II
HA
i
ii
iii
iv
v
B
U87MG-HA-JICD1
DAPI
Human MHC class II
HA
i
ii
iii
iv
v

Supplementary Fig. S3 Immunofluorescence of mouse whole brain section representing invasive tumor margin induced by JICD1, Related to Fig. 1

Immunofluorescence showing the mouse whole-brain section after intracranial injections of U87MG control (A) and HA-JICD1-overexpressing cells (B). White squares indicate the tumor margin regions in Figure 1E. Scale bar = 1000 μm.

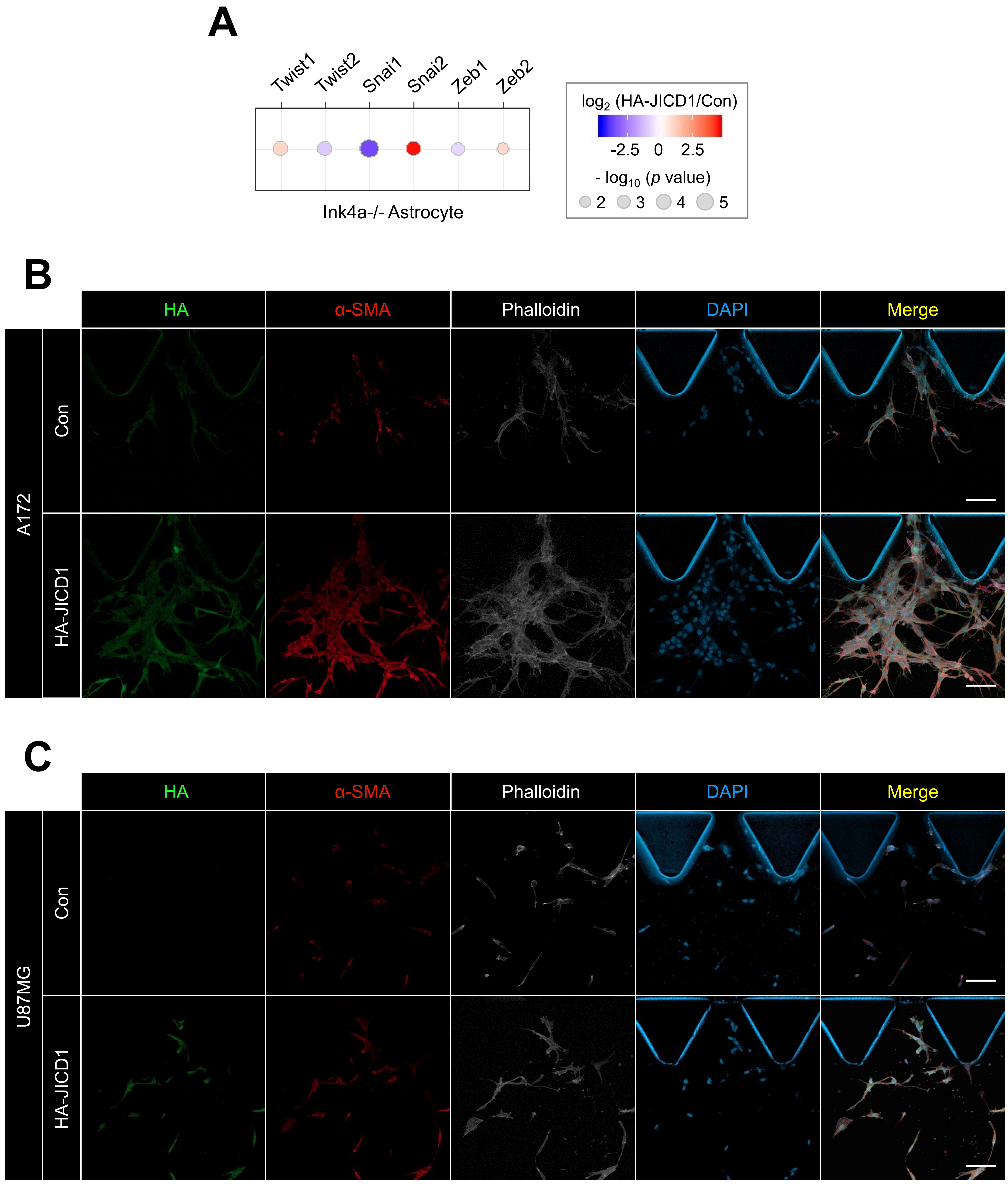

Supplementary Fig. S4 EMT-TFs and marker expression in control and JICD1-overexpressing cells, Related to Fig. 2

(A) A heatmap showing the mRNA expression of canonical EMT-TFs in control and HA-JICD1-overexpressing Ink4a/Arf$^{-/-}$ astrocytes.

(B-C) The expression of α-SMA in HA-JICD1-overexpressing cells. Immunofluorescence showing α-SMA expression of migrated cells in A172 (B) and U87MG (C) control and HA-JICD1 overexpressing cells using 3D cell culture chips. Scale bar = 100 µm.

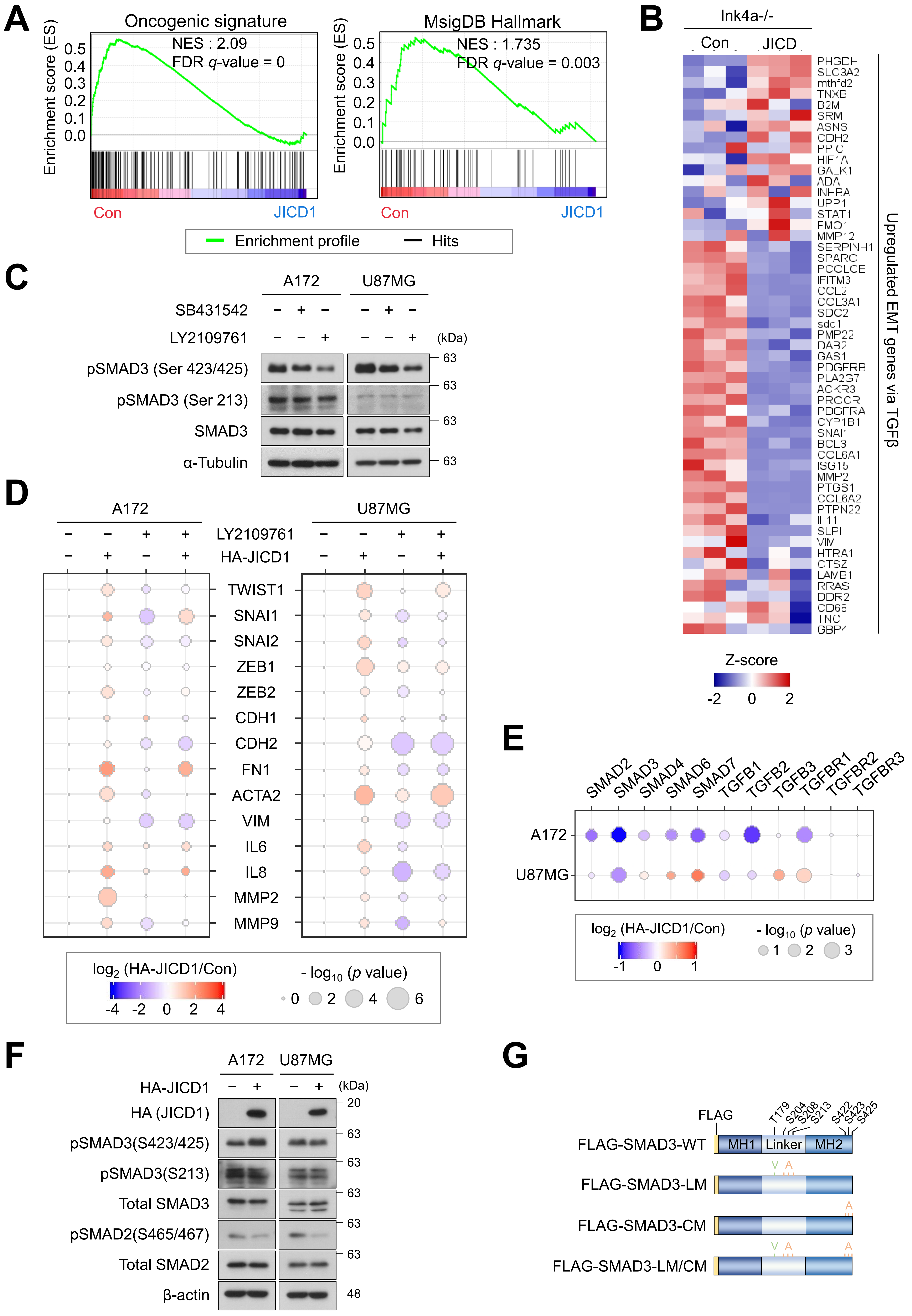

A
Oncogenic signature
NES : 2.09
FDR q-value = 0
Enrichment score (ES)
Con
JICD1
MsigDB Hallmark
NES : 1.735
FDR q-value = 0.003
Enrichment profile
Hits
B
Ink4a-/-
Con
JICD
PHGDH
SLC3A2
mthfd2
TNXB
B2M
SRM
ASNS
CDH2
PPIC
HIF1A
GALK1
ADA
INHBA
UPP1
STAT1
FMO1
MMP12
SERPINH1
SPARC
PCOLCE
IFITM3
CCL2
COL3A1
SDC2
sdc1
PMP22
DAB2
GAS1
PDGFRB
PLA2G7
ACKR3
PROCR
PDGFRA
CYP1B1
SNAI1
BCL3
COL6A1
ISG15
MMP2
PTGS1
COL6A2
PTPN22
IL11
SLPI
VIM
HTRA1
CTSZ
LAMB1
RRAS
DDR2
CD68
TNC
GBP4
Upregulated EMT genes via TGFβ
Z-score
-2 0 2
C
A172
U87MG
SB431542
LY2109761
(kDa)
pSMAD3 (Ser 423/425)
pSMAD3 (Ser 213)
SMAD3
α-Tubulin
63
D
A172
U87MG
LY2109761
HA-JICD1
TWIST1
SNAI1
SNAI2
ZEB1
ZEB2
CDH1
CDH2
FN1
ACTA2
VIM
IL6
IL8
MMP2
MMP9
log2 (HA-JICD1/Con)
-4 -2 0 2 4
- log10 (p value)
0 2 4 6
E
SMAD2
SMAD3
SMAD4
SMAD6
SMAD7
TGFB1
TGFB2
TGFB3
TGFBR1
TGFBR2
TGFBR3
A172
U87MG
log2 (HA-JICD1/Con)
-1 0 1
- log10 (p value)
1 2 3
F
A172
U87MG
HA-JICD1
(kDa)
HA (JICD1)
pSMAD3(S423/425)
pSMAD3(S213)
Total SMAD3
pSMAD2(S465/467)
Total SMAD2
β-actin
20
63
48
G
FLAG
T179
S204
S208
S213
S422
S423
S425
FLAG-SMAD3-WT
MH1
Linker
MH2
FLAG-SMAD3-LM
V
A
FLAG-SMAD3-CM
A
FLAG-SMAD3-LM/CM

Supplementary Fig. S5 Analysis on TGF-β signaling related molecules in control and JICD1-overexpressing cells, Related to Fig. 3

(A) Gene Set Enrichment Analysis demonstrated that two TGF-β signaling gene signatures (oncogenic signature [left] and Broad MsigDB Hallmark [right]) are enriched in control cells than in JICD1-overexpressing Ink4a/Arf$^{-/-}$ astrocytes.

(B) A heatmap showing the mRNA expression of upregulated EMT genes via TGF-β in control and JICD1-overexpressing Ink4a/Arf$^{-/-}$ astrocytes.

(C) Phosphorylation status of SMAD3 in A172 and U87MG cells with SB431542 and LY2109761.

(D) mRNA expression of EMT-related genes in A172 and U87MG control and HA-JICD1-overexpressing cells following LY2109761 treatment.

(E) mRNA expression of key TGF-β signaling factors in A172 and U87MG control and HA-JICD1-overexpressing cells.

(F) Phosphorylation status of SMAD2 and SMAD3 in A172 and U87MG control and HA-JICD1-overexpressing cells.

(G) Schematic diagram representing SMAD3 mutants (WT: Wild-type, LM: Linker mutation, CM: C-tail mutation).

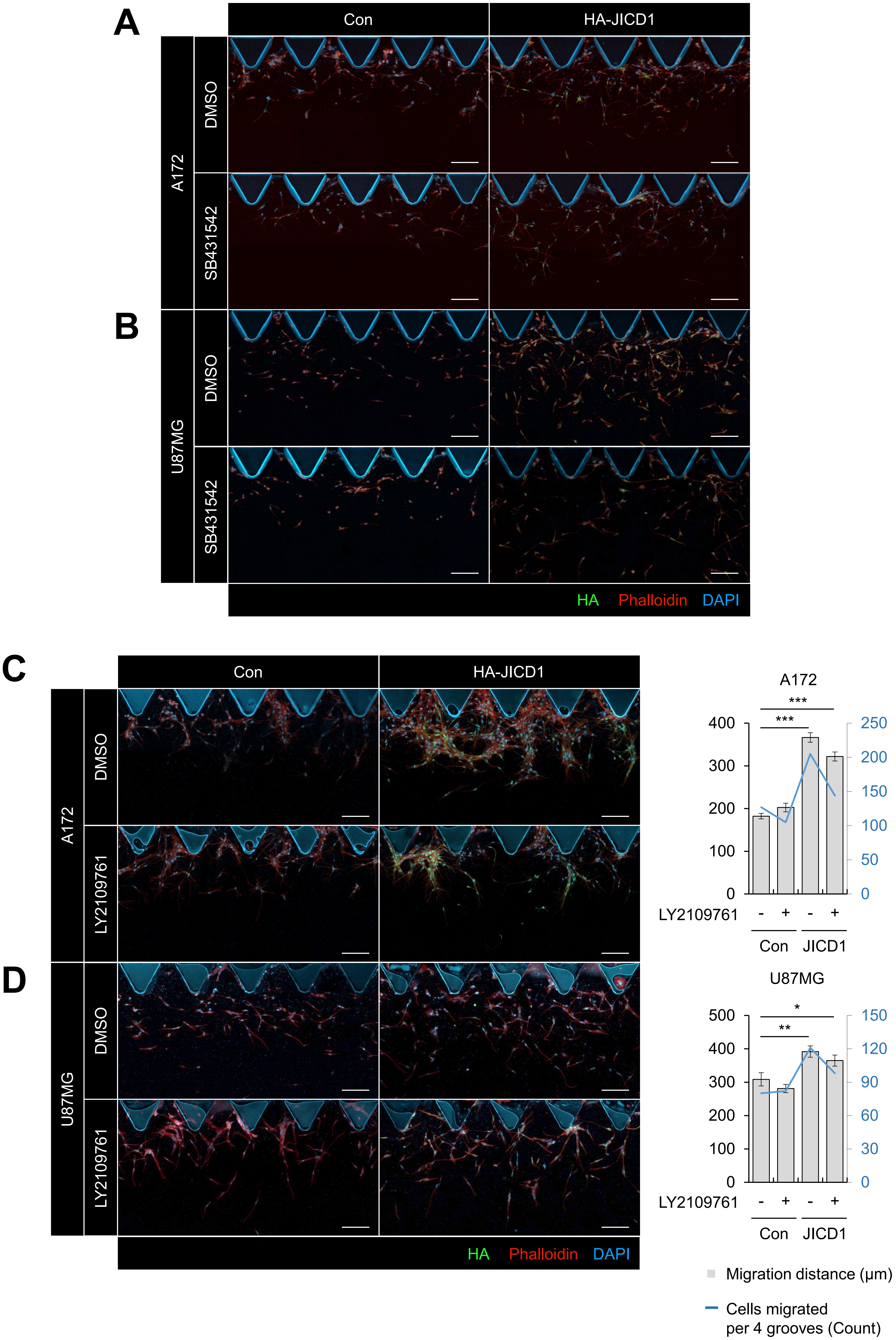
A
Con
HA-JICD1
A172
DMSO
SB431542
B
U87MG
DMSO
SB431542
HA Phalloidin DAPI
C
Con
HA-JICD1
A172
DMSO
LY2109761
D
U87MG
DMSO
LY2109761
HA Phalloidin DAPI
A172
***
***
400
300
200
100
0
250
200
150
100
50
0
LY2109761 - + - +
Con JICD1
U87MG
*
**
500
400
300
200
100
0
150
120
90
60
30
0
LY2109761 - + - +
Con JICD1
Migration distance (μm)
Cells migrated per 4 grooves (Count)

Supplementary Fig. S6 3D cell migration assay of JICD1-overexpressing cells following treatment with SB43154 or LY2109761, Related to Fig. 3

(A, B) Immunofluorescence results show the HA-JICD1 expression of migrated cells in (A) A172 and (B) U87MG control and HA-JICD1-overexpressing cells treated with SB431542 (A172: 10 μM, U87MG: 20 μM). Scale bar = 200 μm.

(C, D) Immunofluorescence results show the HA-JICD1 expression of migrated cells in (C) A172 and (D) U87MG control and HA-JICD1-overexpressing cells treated with LY2109761 (A172, U87MG: 5 μM). Scale bar = 200 μm. Bar graph representing the average migration distance (black, left axis) and number of migrated cells per four grooves (blue, right axis) in A172 and U87MG control and HA-JICD1-overexpressing cells with LY2109761 treatment. ***$p < 0.001$.

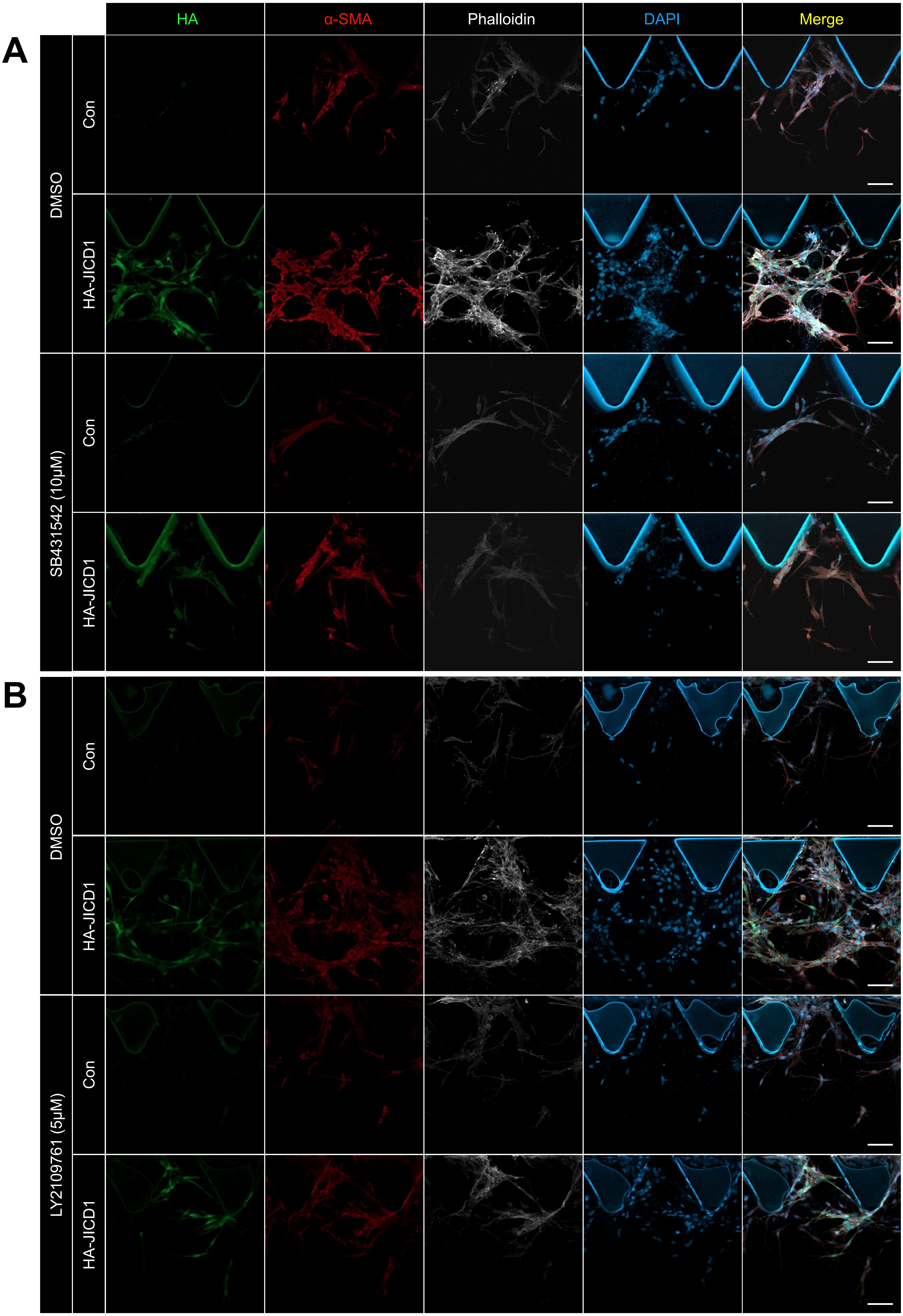

A
B
HA
α-SMA
Phalloidin
DAPI
Merge
DMSO
SB431542 (10μM)
LY2109761 (5μM)
Con
HA-JICD1

Supplementary Fig. S7 α-SMA expression of SB431542- or LY2109761-treated A172 control and JICD1-overexpressing cells on 3D cell culture chips, Related to Fig. 3

Immunofluorescence showing α-SMA expression in migrated A172 control and HA-JICD1-overexpressing cells treated with (A) SB431542 and (B) LY2109761 in 3D cell culture chips. Scale bar = 100 μm.

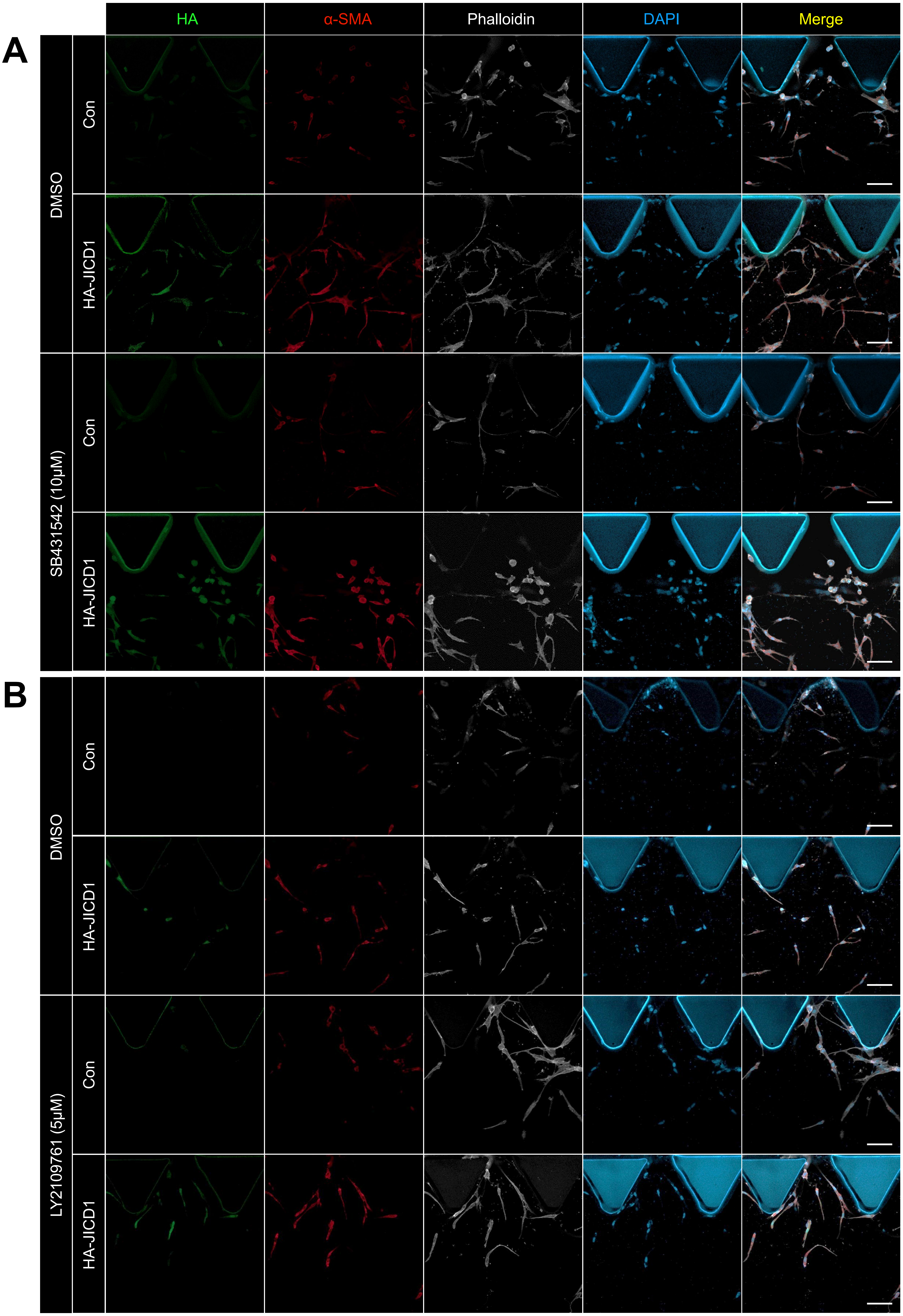
A
B
HA
α-SMA
Phalloidin
DAPI
Merge
DMSO
SB431542 (10μM)
LY2109761 (5μM)
Con
HA-JICD1

Supplementary Fig. S8 α-SMA expression of SB431542- or LY2109761-treated U87MG control and JICD1-overexpressing cells on 3D cell culture chips, Related to Fig. 3

Immunofluorescence showing α-SMA expression in migrated U87MG control and HA-JICD1-overexpressing cells treated with (A) SB431542 and (B) LY2109761 in 3D cell culture chips. Scale bar = 100 μm.

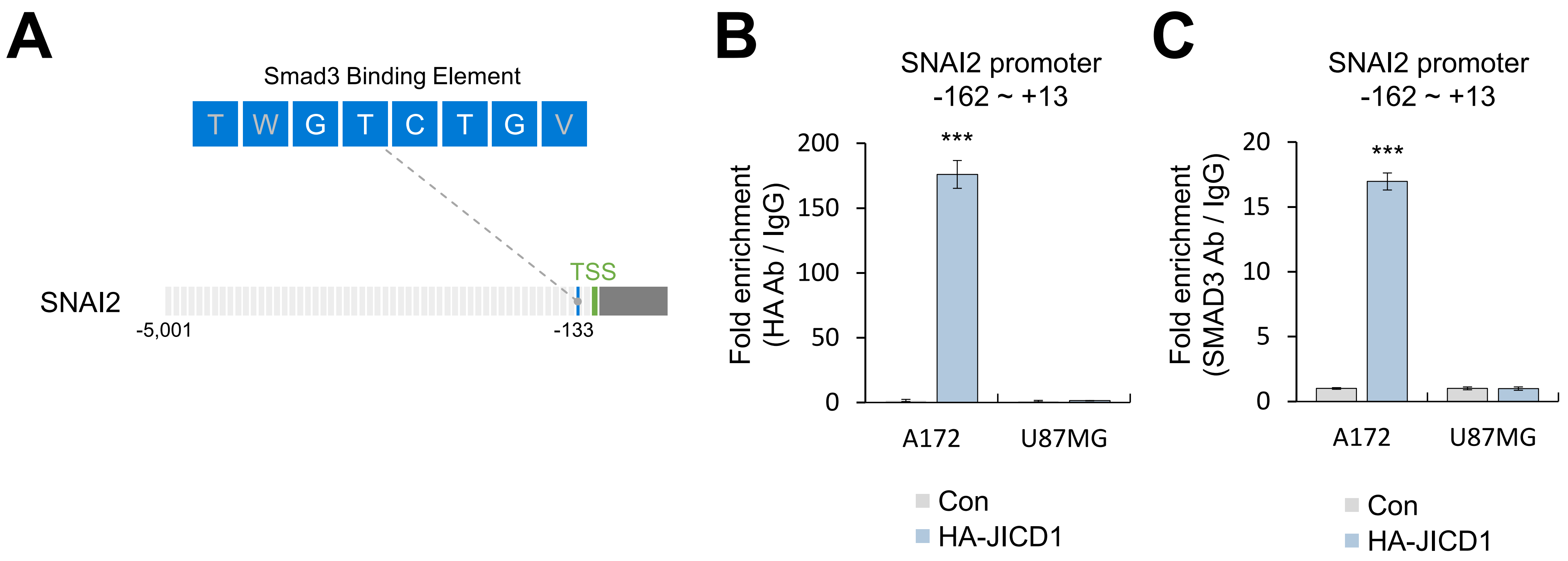

Supplementary Fig. S9 ChIP assay of EMT-TFs upregulated by JICD1 transcriptional complex, Related to Fig. 4

(A) A schematic diagram of a SMAD3 binding element (TWGTCTGV) in the *SNAI2* promoter.

(B-C) ChIP-PCR analysis of HA-JICD1 (B) and SMAD3 (C) engagement with the SMAD3 binding motif at the *SNAI2* promoter in A172 and U87MG control and HA-JICD1-overexpressing cells. $^{***}p < 0.001$.

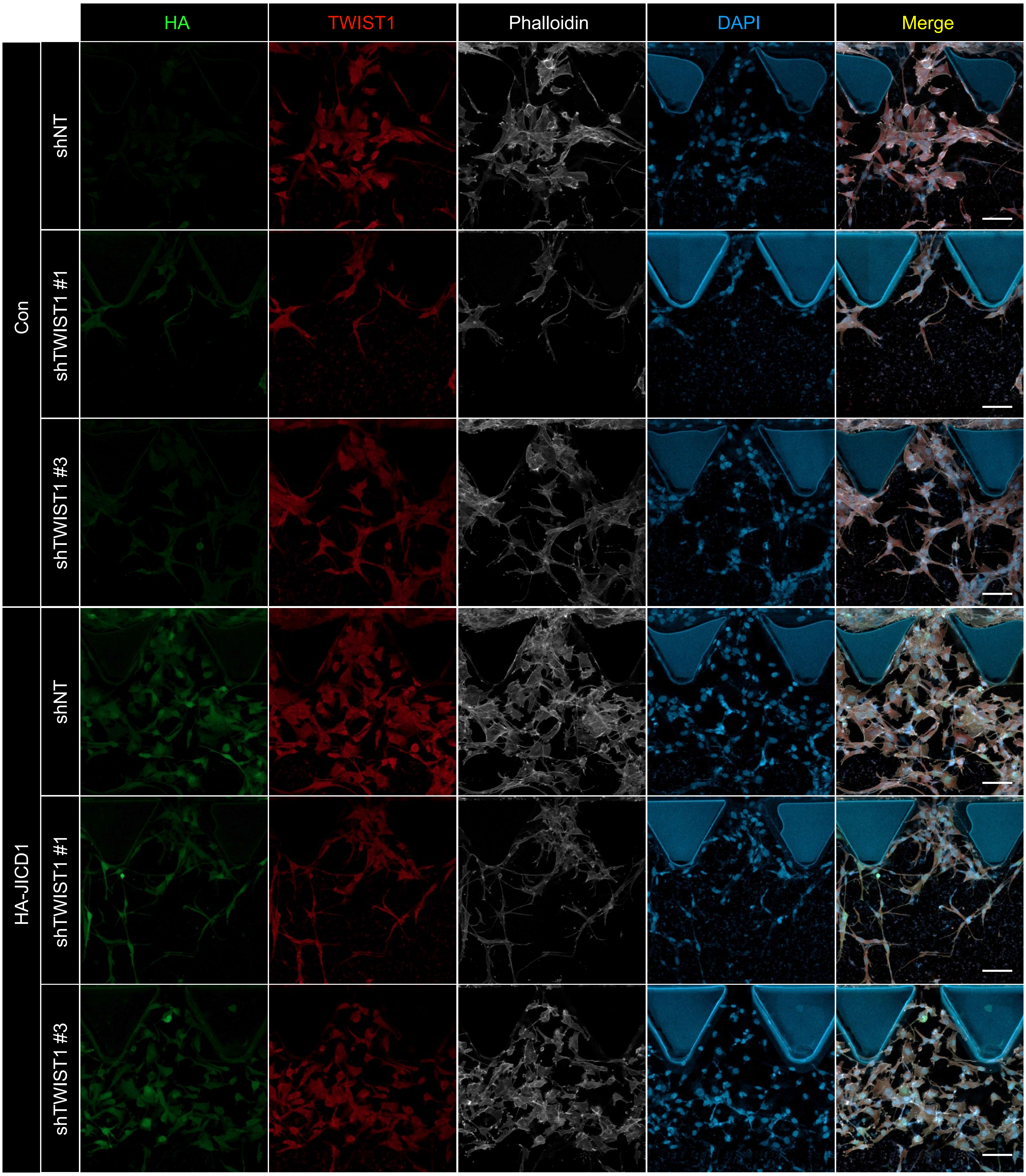

Supplementary Fig. S10 TWIST1 expression in TWIST1 silenced-A172 control and JICD1-overexpressing cells on 3D cell culture chips, Related to Fig. 4

Immunofluorescence showing TWIST1 expression of migrated cells in A172 control and HA-JICD1 overexpressing cells with shTWIST1 using 3D cell culture chips. Scale bar = 100 µm.

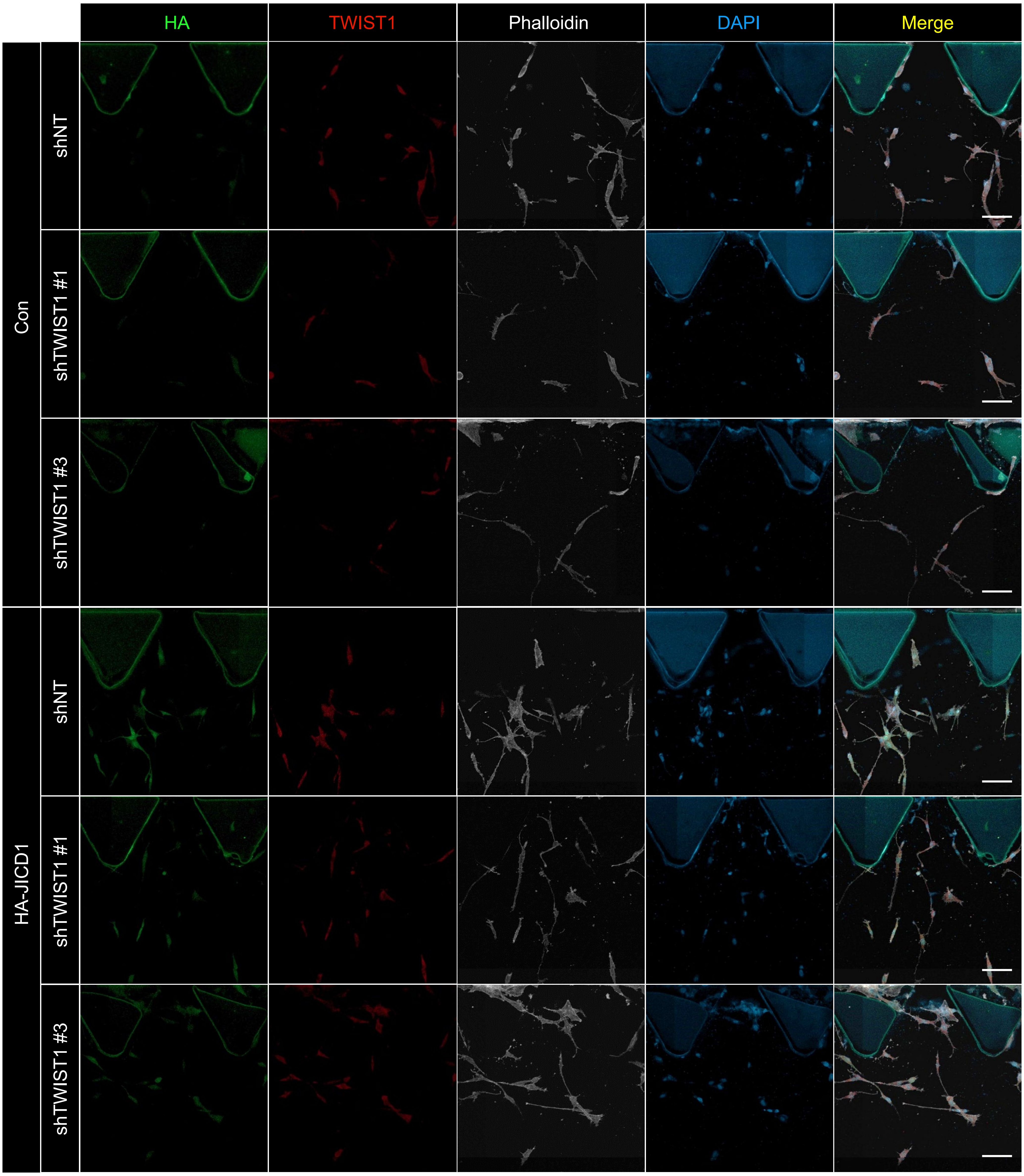

Supplementary Fig. S11 TWIST1 expression in TWIST1 silenced-U87MG control and JICD1-overexpressing cells on 3D cell culture chips, Related to Fig. 4

Immunofluorescence showing TWIST1 expression of migrated cells in U87MG control and HA-JICD1 overexpressing cells with shTWIST1, using 3D cell culture chips. Scale bar = 100 μm.

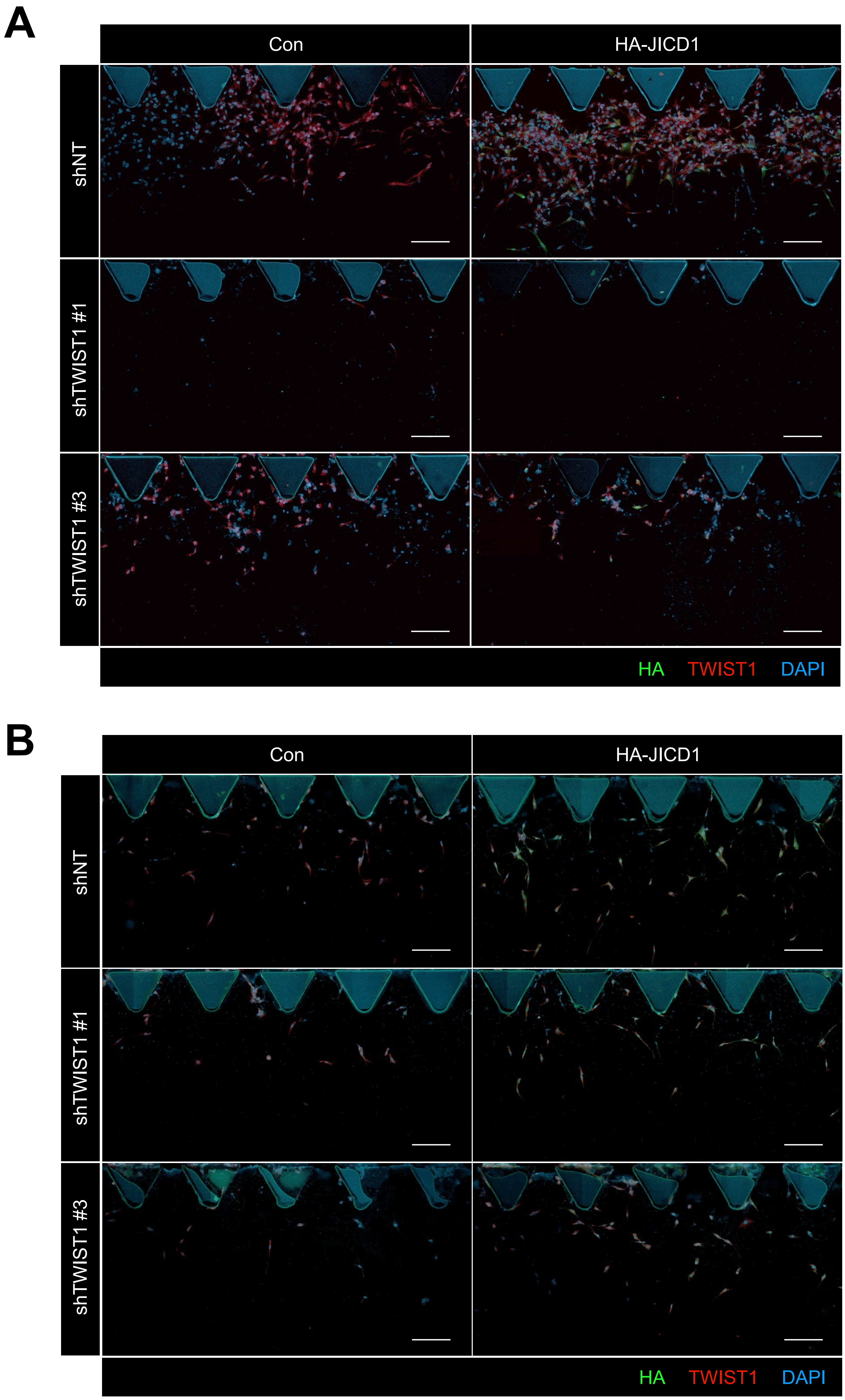

Supplementary Fig. S12 3D cell migration assay of control and JICD1-overexpressing cells after TWIST1 knockdown, Related to Fig. 4

Immunofluorescence showing HA-JICD1 expression of migrated cells in A172 (A) and U87MG (B) control and HA-JICD1-overexpressing cells with TWIST1 knockdown, using 3D cell culture chips. Scale bar = 200 μm.

A

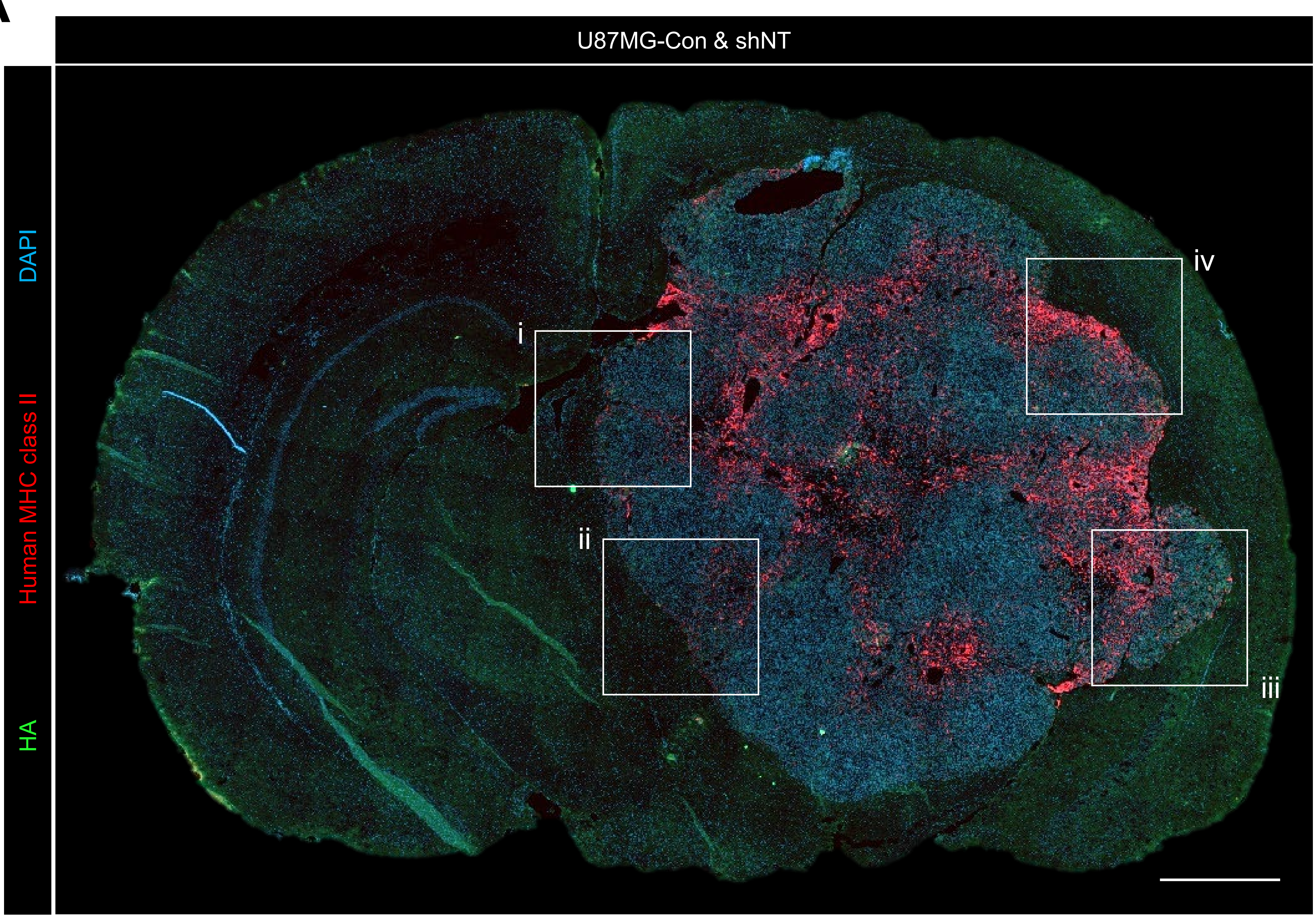
U87MG-Con & shNT
DAPI
Human MHC class II
HA
i
ii
iii
iv

B

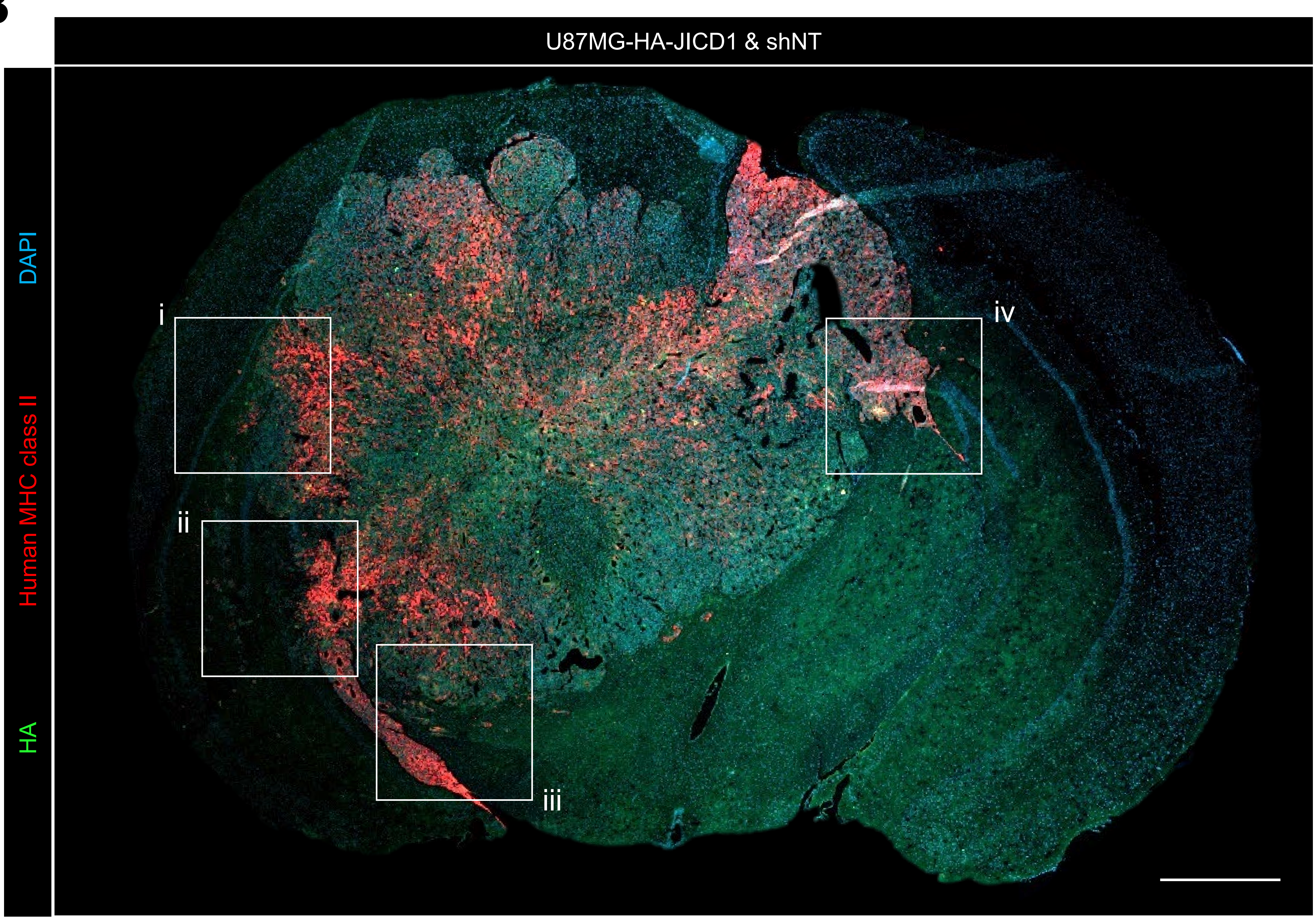
U87MG-HA-JICD1 & shNT
DAPI
Human MHC class II
HA
i
ii
iii
iv

C

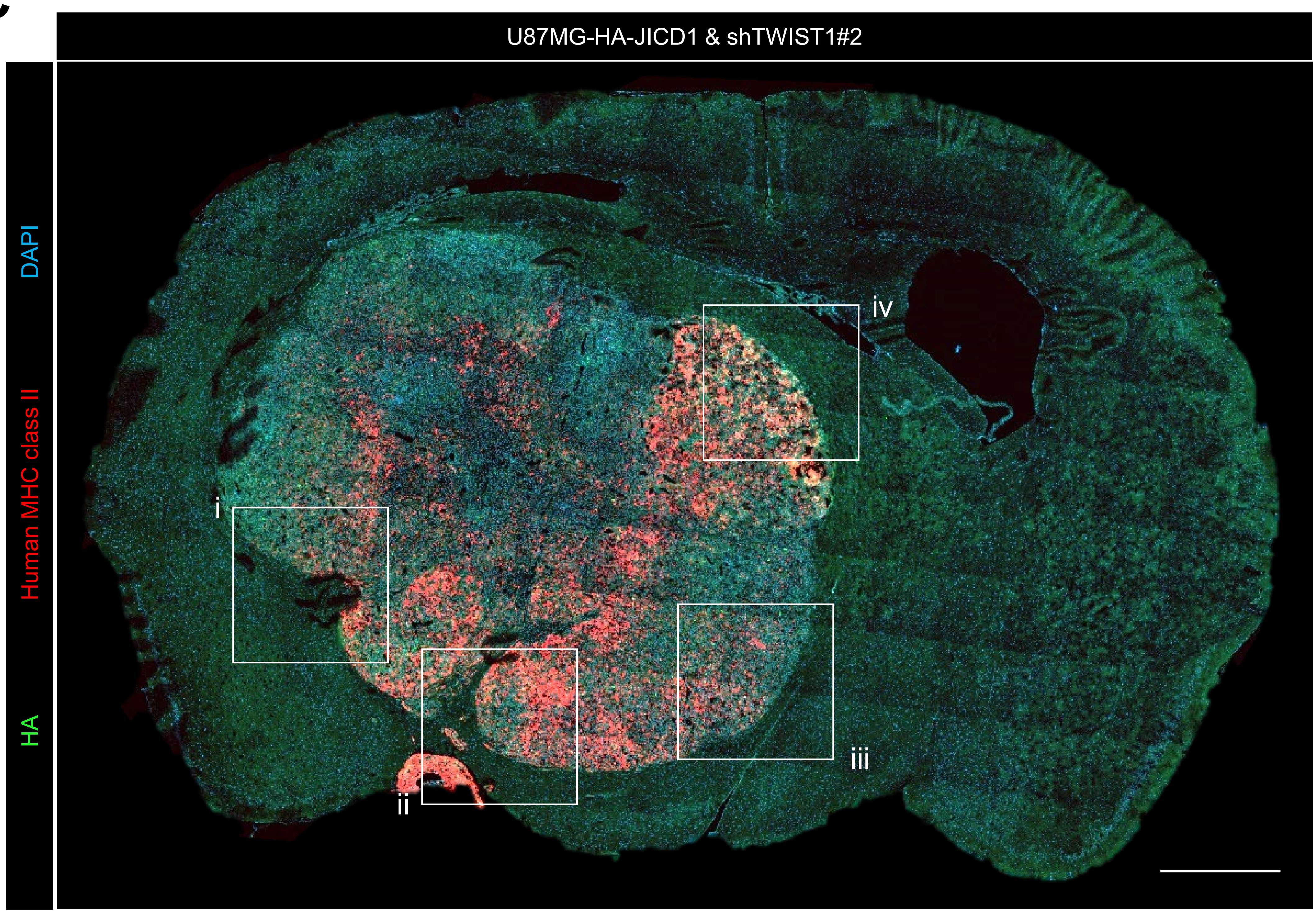

Supplementary Fig. S13 Immunofluorescence of mouse whole-brain section showing invasive tumor margin, Related to Fig. 5

Immunofluorescence showing mouse whole-brain sections after intracranial injection of U87MG control (A), HA-JICD1-overexpressing cells without (B) and with TWIST1 knockdown (C). White squares indicate the tumor margin regions in Figure 5C. Scale bar = 1000 μm.

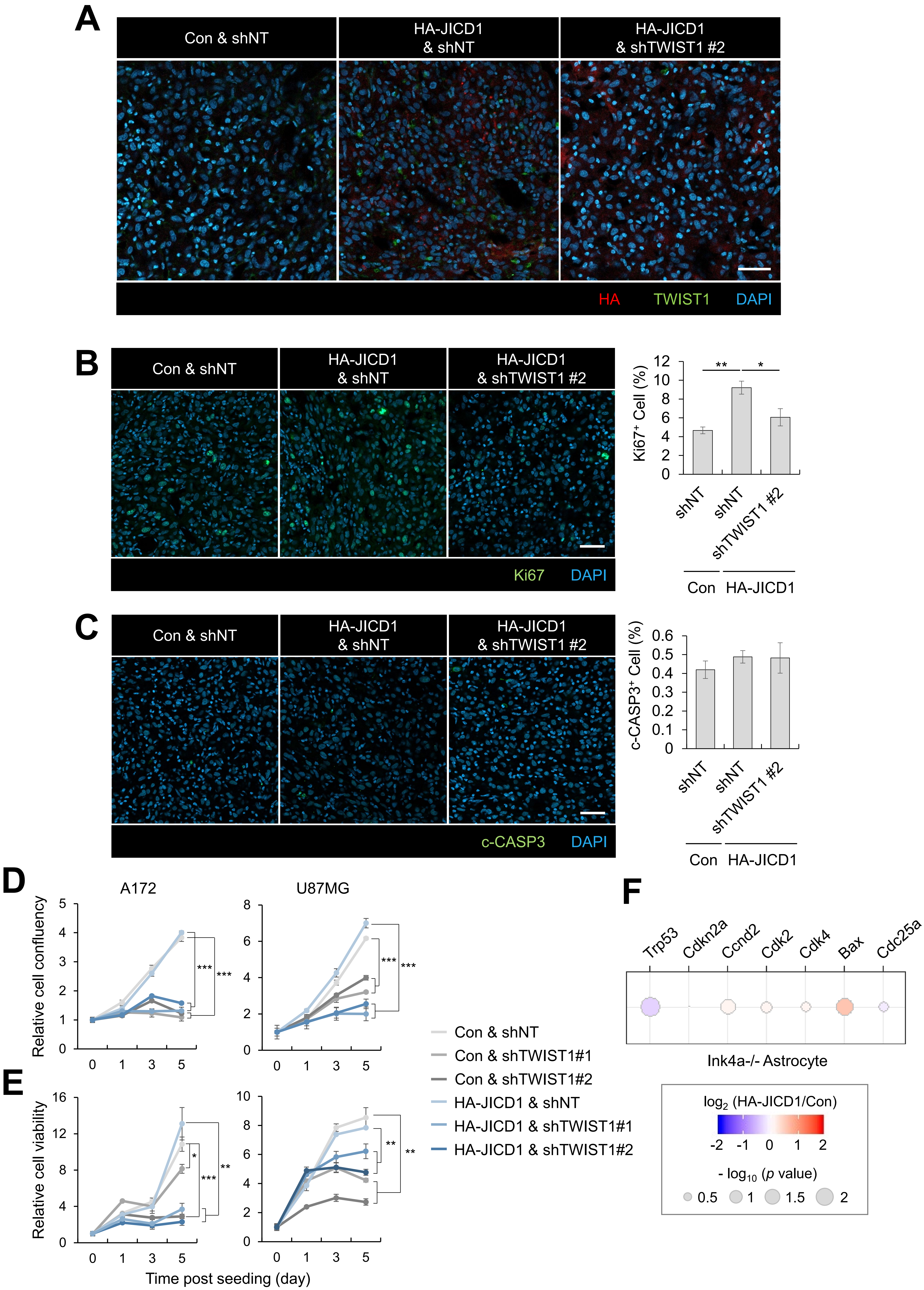
A
Con & shNT
HA-JICD1 & shNT
HA-JICD1 & shTWIST1 #2
HA
TWIST1
DAPI
B
Con & shNT
HA-JICD1 & shNT
HA-JICD1 & shTWIST1 #2
Ki67
DAPI
Ki67+ Cell (%)
12
10
8
6
4
2
0
**
*
shNT
shNT
shTWIST1 #2
Con
HA-JICD1
C
Con & shNT
HA-JICD1 & shNT
HA-JICD1 & shTWIST1 #2
c-CASP3
DAPI
c-CASP3+ Cell (%)
0.6
0.5
0.4
0.3
0.2
0.1
0
shNT
shNT
shTWIST1 #2
Con
HA-JICD1
D
A172
U87MG
Relative cell confluency
***
***
E
Relative cell viability
*
***
**
Time post seeding (day)
0
1
3
5
Con & shNT
Con & shTWIST1#1
Con & shTWIST1#2
HA-JICD1 & shNT
HA-JICD1 & shTWIST1#1
HA-JICD1 & shTWIST1#2
F
Trp53
Cdkn2a
Ccnd2
Cdk2
Cdk4
Bax
Cdc25a
Ink4a-/- Astrocyte
log2 (HA-JICD1/Con)
-2
-1
0
1
2
- log10 (p value)
0.5
1
1.5
2

Supplementary Fig. S14 Histological characteristics of JICD1-overexpressing cells with or without TWIST1 knockdown, Related to Fig. 5

(A) Immunofluorescence showing TWIST1 expression in mouse brain sections after intracranial injections of U87MG control and HA-JICD1-overexpressing cells with TWIST1 knockdown. Scale bar = 100 µm.

(B-C) Immunofluorescence images for Ki67 (B) and cleaved caspase3 (c-CASP3) (C). Scale bar = 100 µm. The bar graphs represent means ± SEM.

(D) Relative cell confluency of A172 and U87MG control and HA-JICD1-overexpressing cells with shTWIST1. ***$p < 0.001$.

(E) Relative cell viability of A172 and U87MG control and HA-JICD1-overexpressing cells with shTWIST1. *$p<0.05$, **$p<0.01$, ***$p < 0.001$.

(F) A heatmap showing the mRNA expression of TWIST1-regulated cell proliferation and cell death gene in control and HA-JICD1-overexpressing Ink4a/Arf$^{-/-}$ astrocytes.

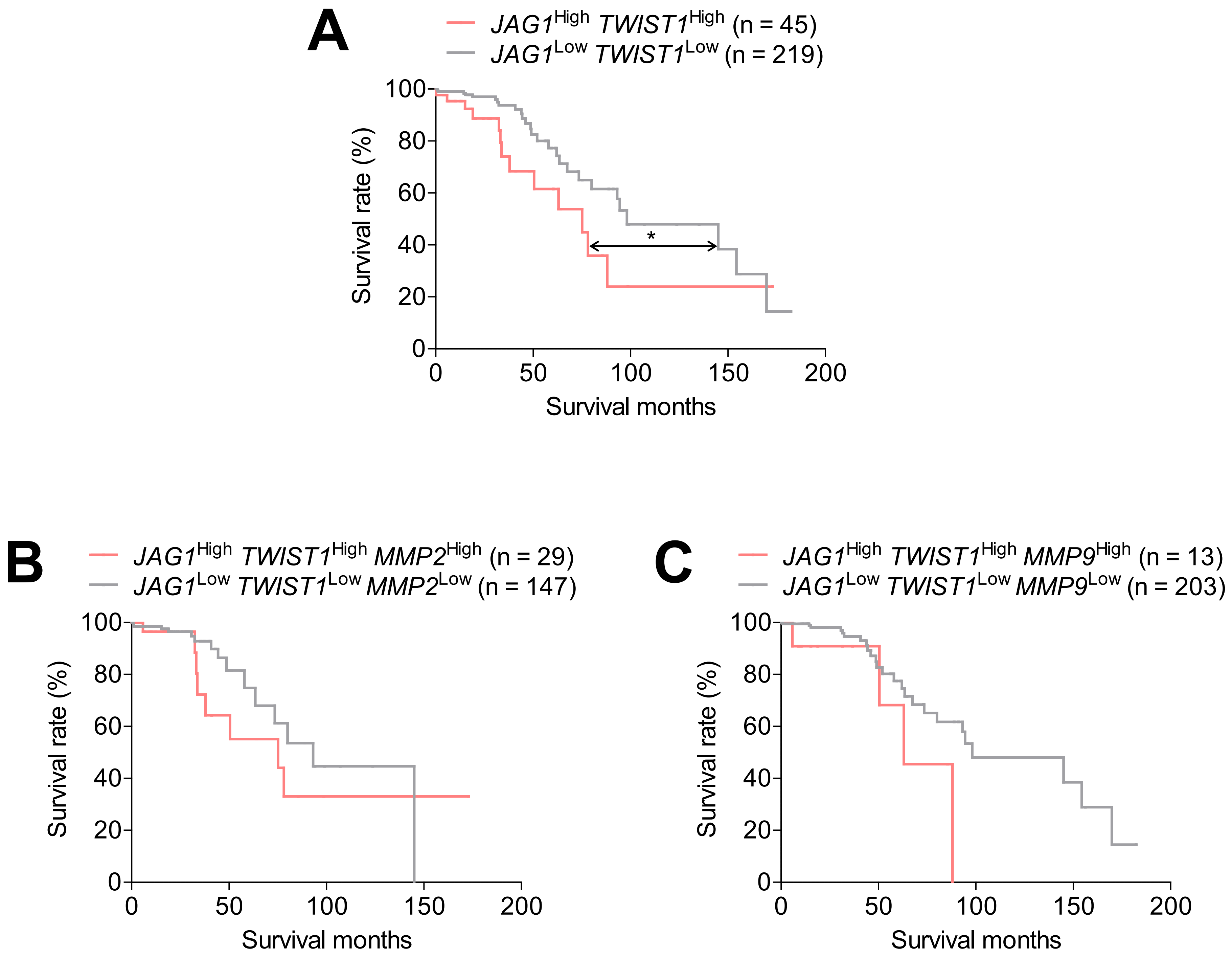

Supplementary Fig. S15 Survival rate of patients with IDH-mutant glioma according to JAG1, TWIST1, MMP2 and MMP9 expression. Related to Fig. 6

(A) Survival rate of patients with IDH-mutant glioma according to JAG1 and TWIST1 expression. The patients were divided into two groups: JAG1-high and TWIST1-high vs JAG1-low and TWIST1-low, based on mRNA expression levels (mean ± SEM). *P*-values were calculated using a log-rank (Mantel-Cox) test. $*p < 0.05$.

(B, C) Survival rate of patients with IDH-mutant glioma according to JAG1, TWIST1, MMP2, and MMP9 expression. The patients were divided into two groups: JAG1-high, TWIST1-high, and MMP2-high vs JAG1-low, TWIST1-low, and MMP2-low (B) and JAG1-high, TWIST1-high, and MMP9-high vs JAG1-low, TWIST1-low, and MMP9-low (C) based on mRNA expression (mean ± SEM). *P*-values were calculated by a log-rank (Mantel-Cox) test.
