# Supplementary material for: Jagged1 intracellular domain/SMAD3 complex transcriptionally regulates TWIST1 to drive glioma invasion
Source: Cell Death Dis. 2023 Dec 13;14(12):822. doi: 10.1038/s41419-023-06356-0 (PMC10719344; doi:10.1038/s41419-023-06356-0)

Original Data File

Original scans of western blots

Fig. 2B

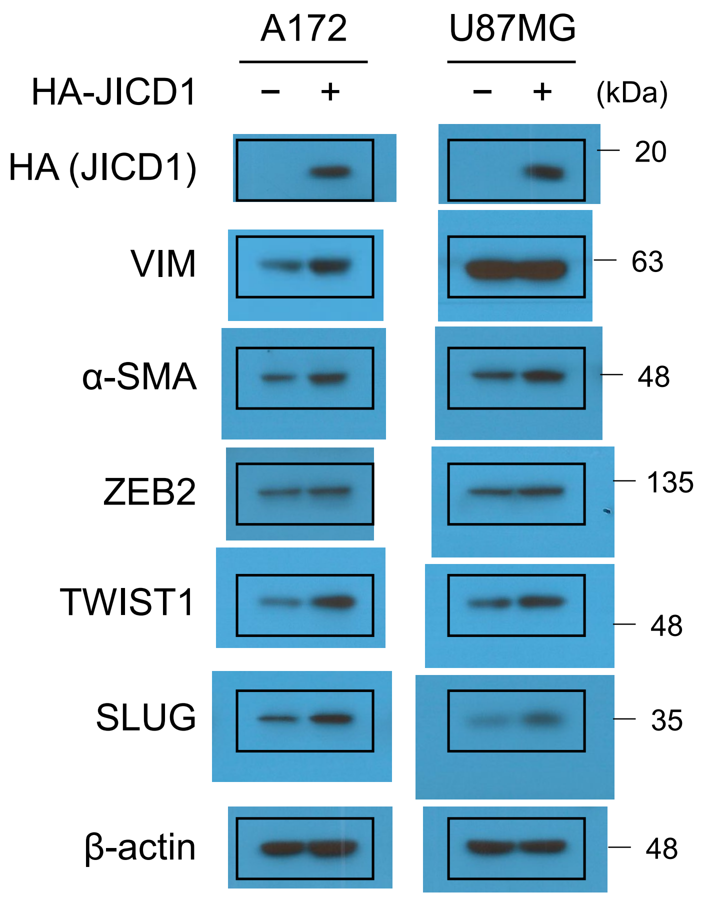

Fig. 2D

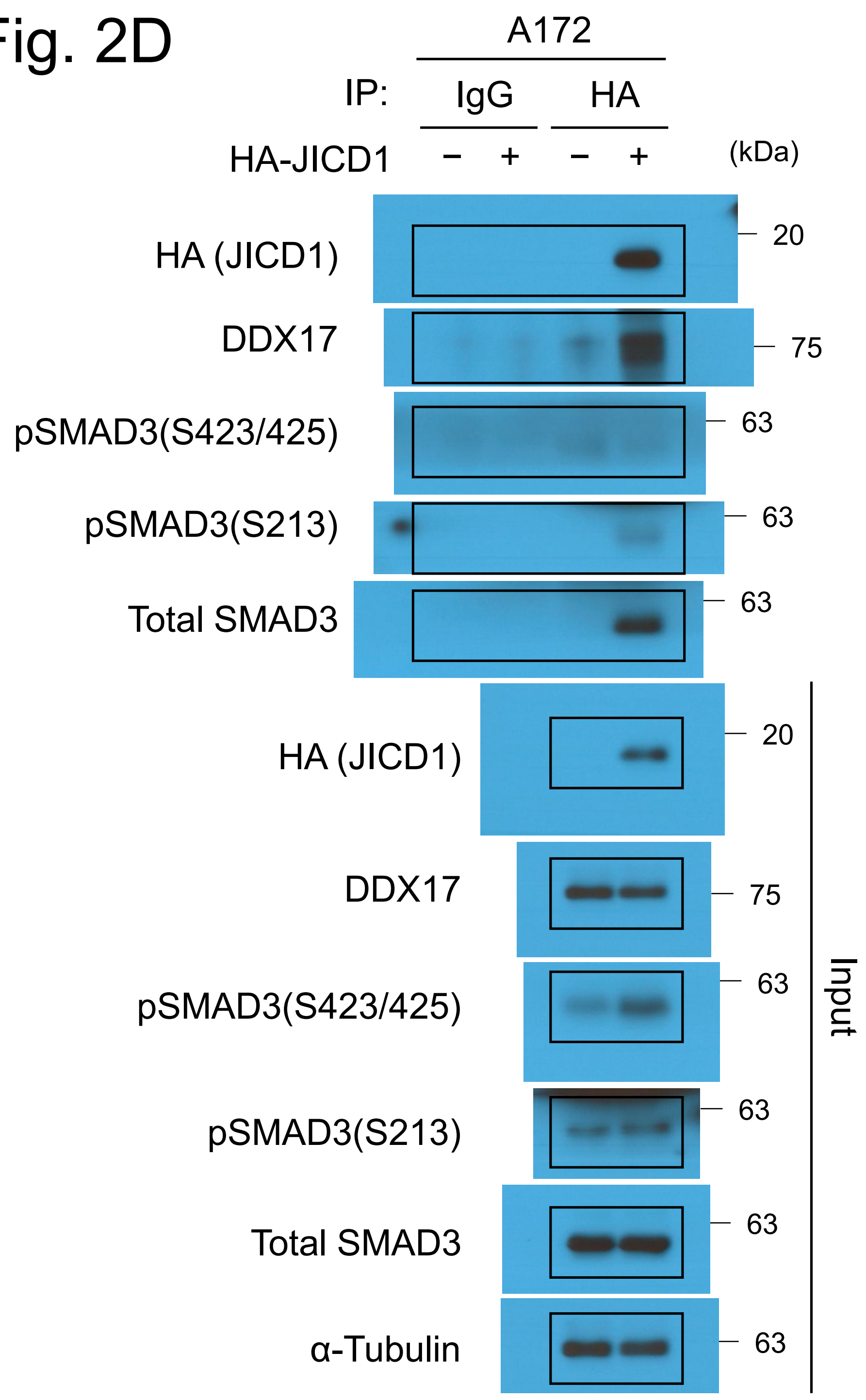

Fig. 2E

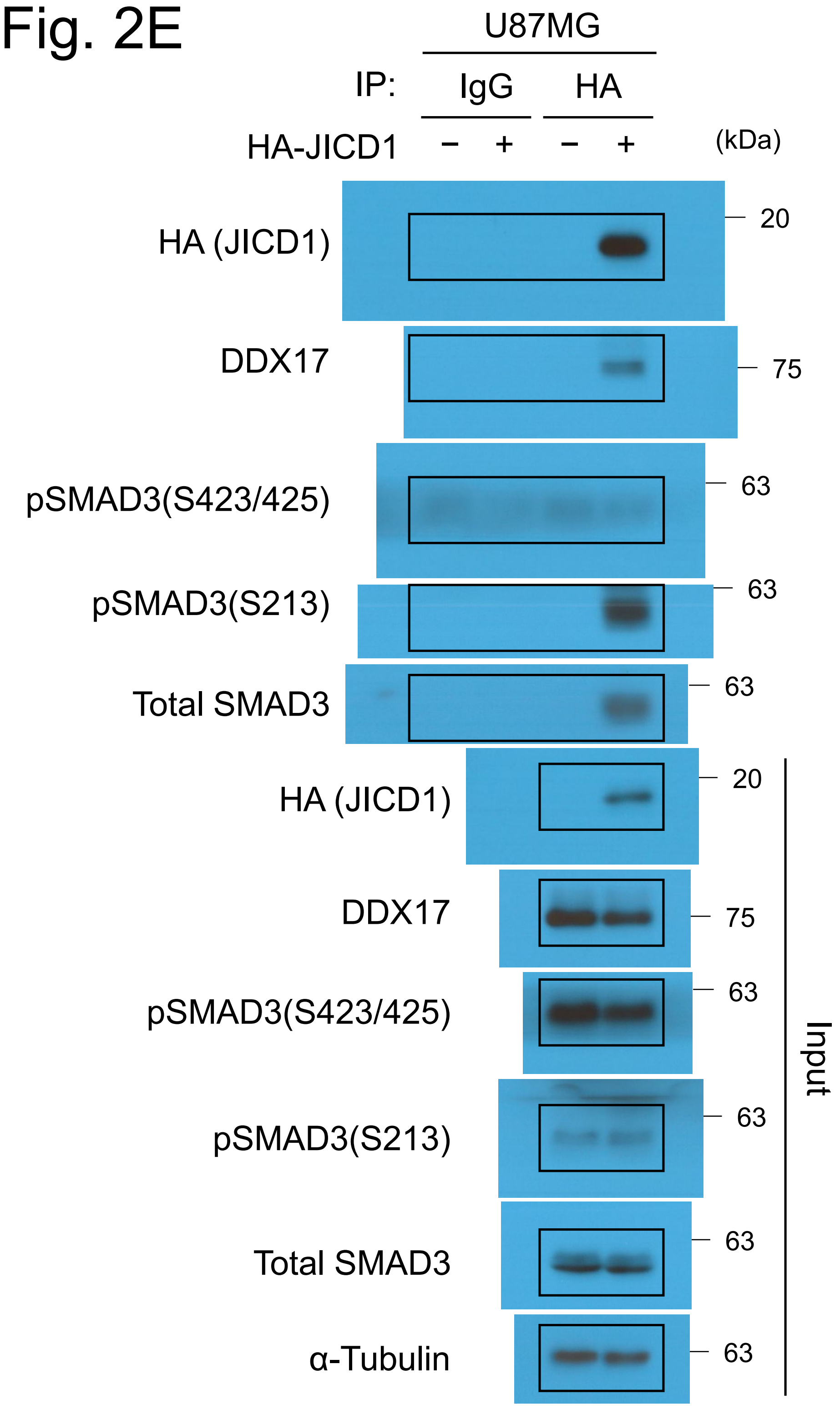

Fig. 3C

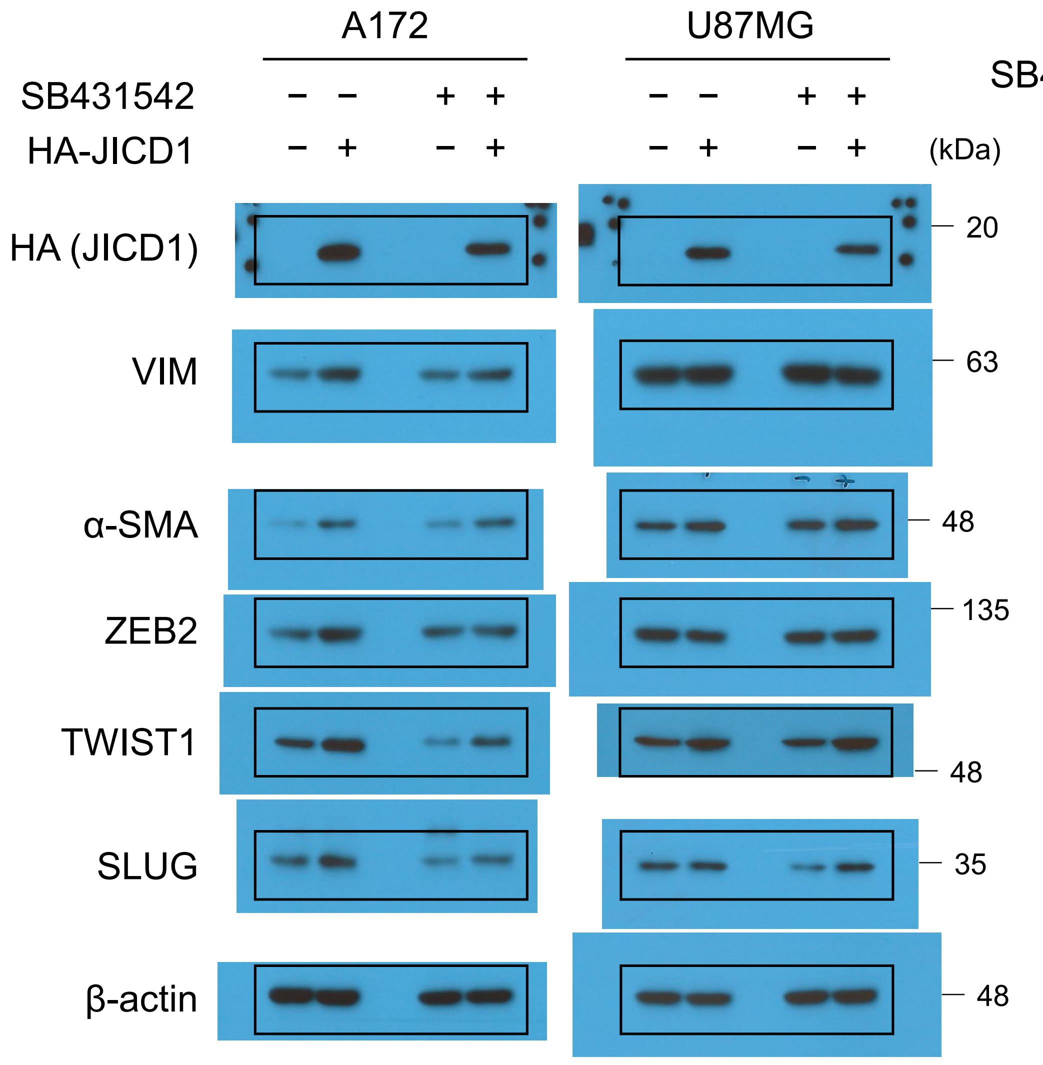

Fig. 3D

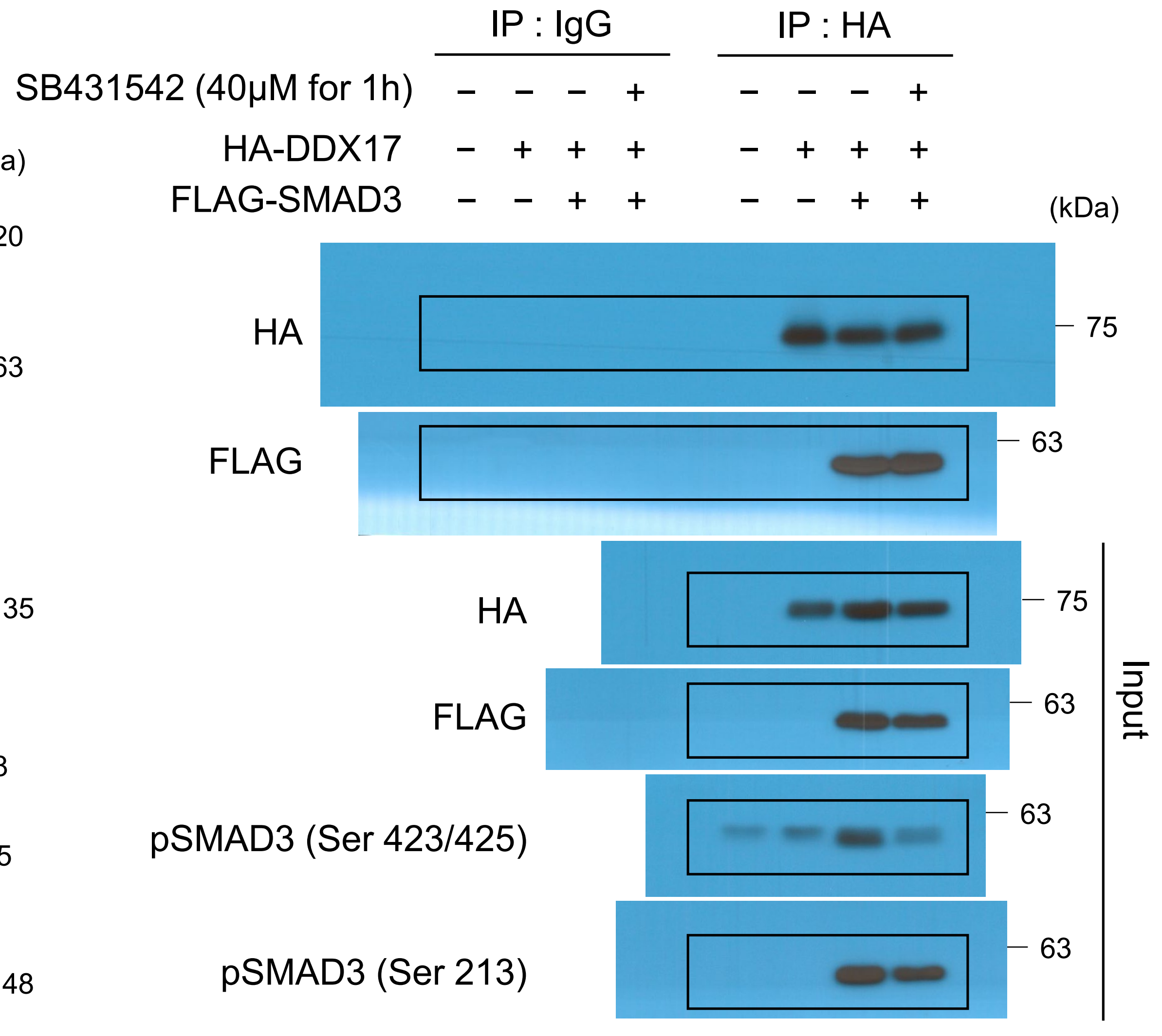

Fig. 3E

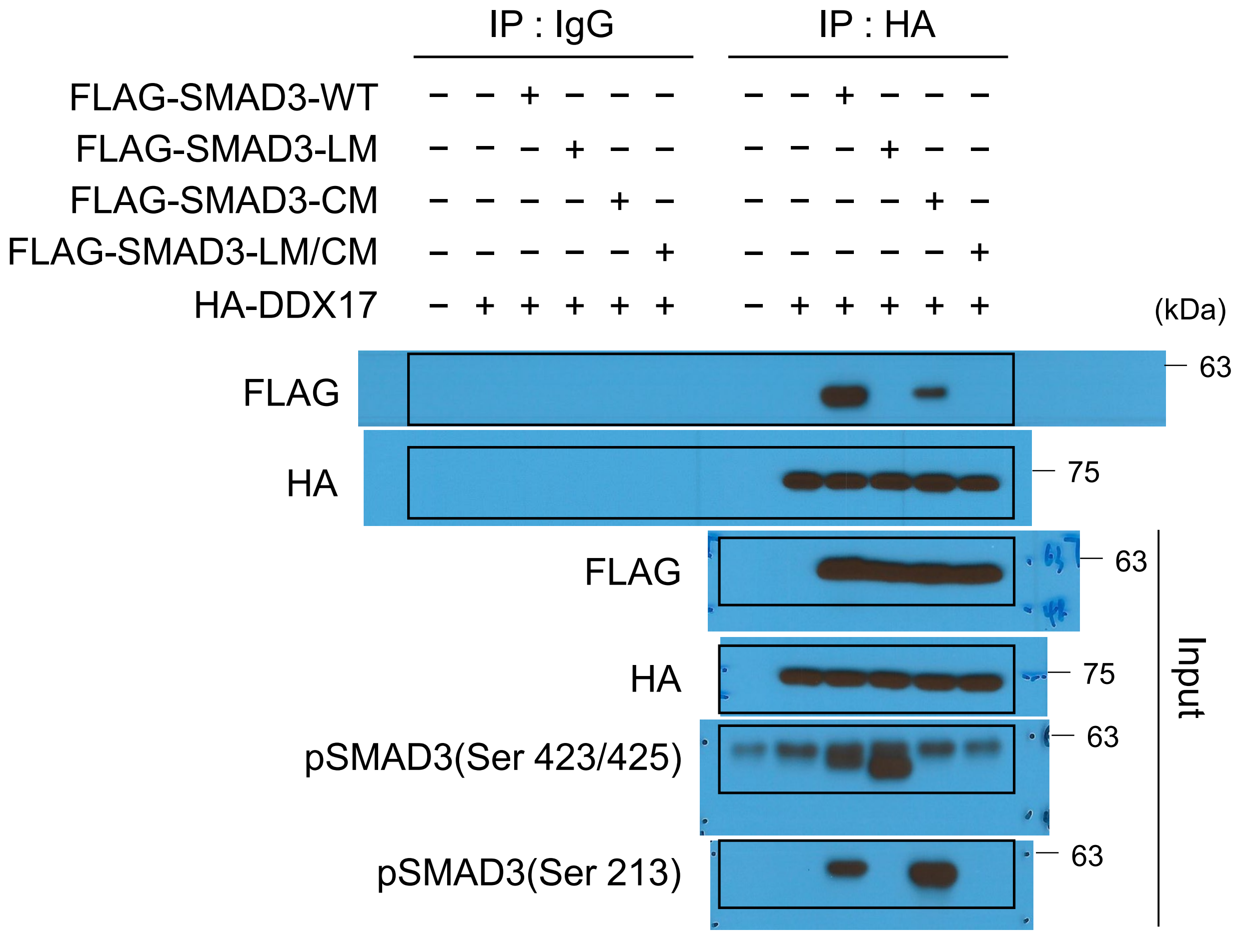

Supplementary fig. 2A

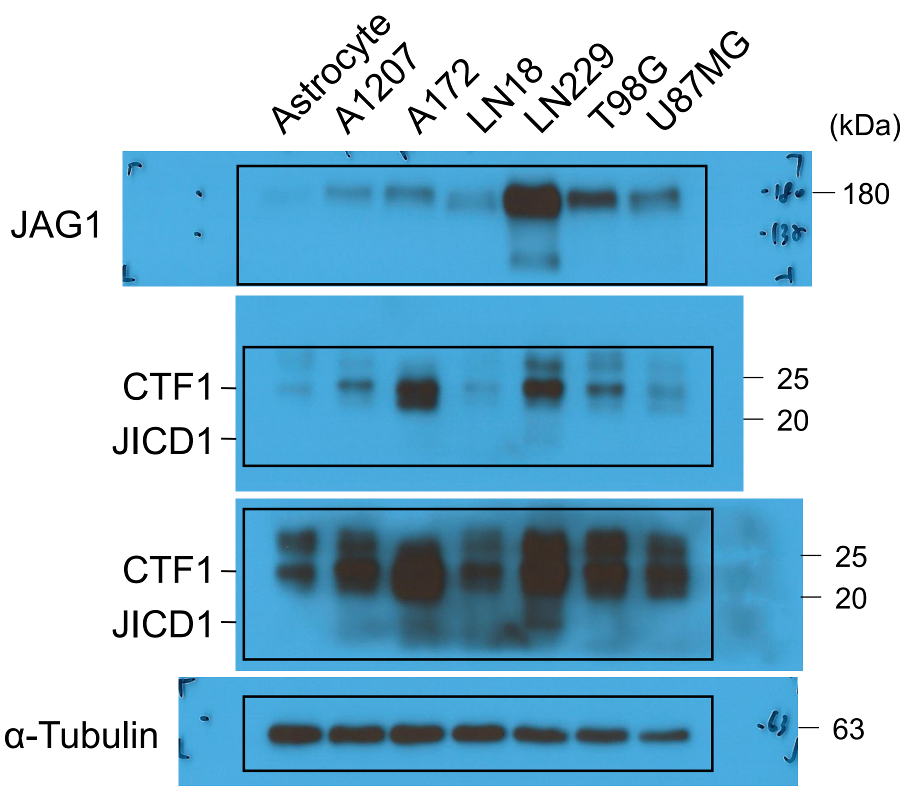

Supplementary fig. 2B

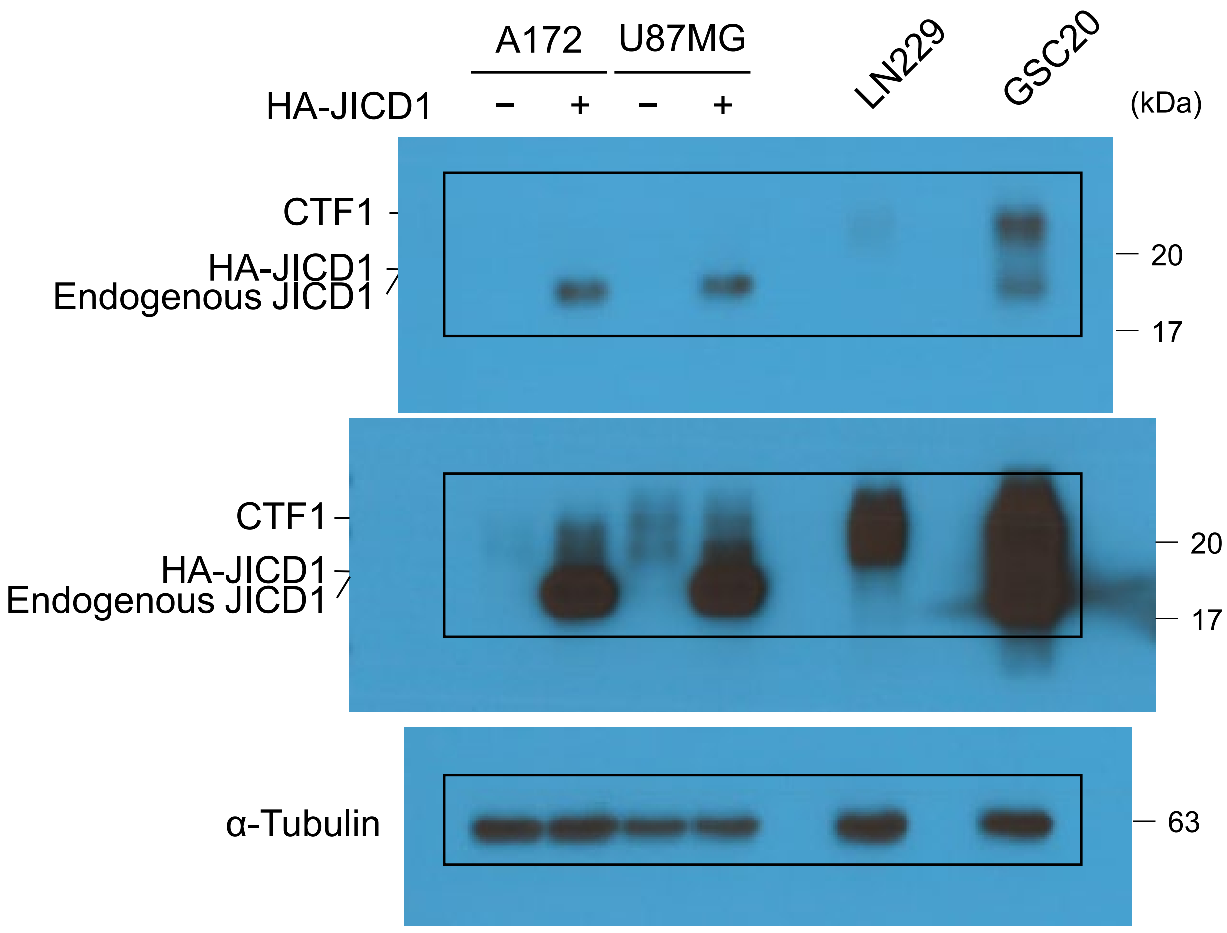

Supplementary fig. 5C

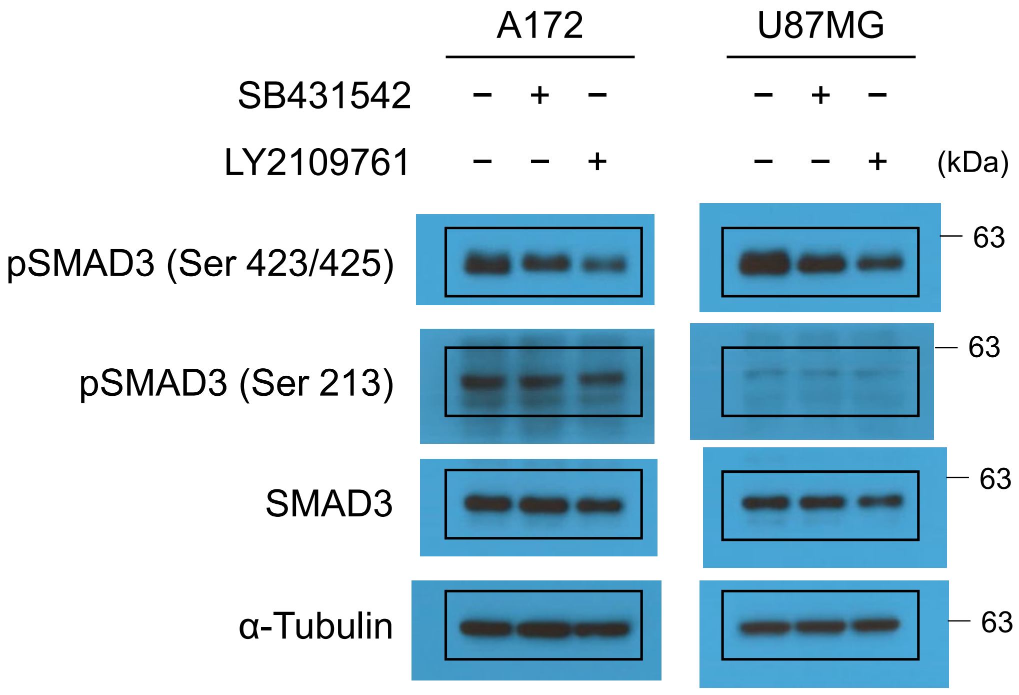

Supplementary fig. 5F

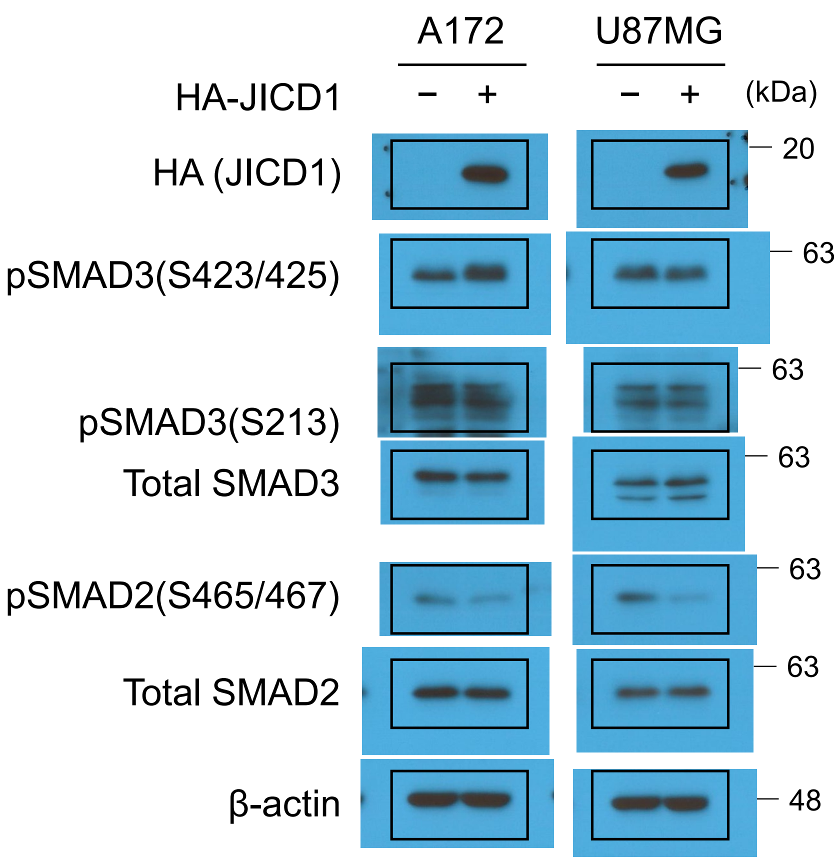

Supplement: Supplementary file 2 — Original Data File [file 41419_2023_6356_MOESM2_ESM.pdf]
